# The ApaH-like phosphatase TbALPH1 is the major mRNA decapping enzyme of trypanosomes

**DOI:** 10.1371/journal.ppat.1006456

**Published:** 2017-06-19

**Authors:** Susanne Kramer

**Affiliations:** Biocenter, University of Würzburg, Am Hubland, Würzburg, Germany; University of California, Los Angeles, UNITED STATES

## Abstract

5’-3’ decay is the major mRNA decay pathway in many eukaryotes, including trypanosomes. After deadenylation, mRNAs are decapped by the nudix hydrolase DCP2 of the decapping complex and finally degraded by the 5’-3’ exoribonuclease. Uniquely, trypanosomes lack homologues to all subunits of the decapping complex, while deadenylation and 5’-3’ degradation are conserved. Here, I show that the parasites use an ApaH-like phosphatase (ALPH1) as their major mRNA decapping enzyme. The protein was recently identified as a novel trypanosome stress granule protein and as involved in mRNA binding. A fraction of ALPH1 co-localises exclusively with the trypanosome 5’-3’ exoribonuclease XRNA to a special granule at the posterior pole of the cell, indicating a connection between the two enzymes. RNAi depletion of ALPH1 is lethal and causes a massive increase in total mRNAs that are deadenylated, but have not yet started 5’-3’ decay. These data suggest that ALPH1 acts downstream of deadenylation and upstream of mRNA degradation, consistent with a function in mRNA decapping. *In vitro* experiments show that recombinant, N-terminally truncated ALHP1 protein, but not a catalytically inactive mutant, sensitises the capped trypanosome spliced leader RNA to yeast Xrn1, but only if an RNA 5’ polyphosphatase is included. This indicates that the decapping mechanism of ALPH1 differs from the decapping mechanism of Dcp2 by leaving more than one phosphate group at the mRNA’s 5’ end. This is the first reported function of a eukaryotic ApaH-like phosphatase, a bacterial-derived class of enzymes present in all phylogenetic super-groups of the eukaryotic kingdom. The substrates of eukaryotic ApaH-like phosphatases are unknown. However, the substrate of the related bacterial enzyme ApaH, diadenosine tetraphosphate, is highly reminiscent of a eukaryotic mRNA cap.

## Author summary

Eukaryotic mRNAs are stabilised by a 5’ cap and one important step in mRNA decay is the removal of this cap by the nudix domain protein Dcp2 of the decapping complex. The decapping complex is highly conserved throughout eukaryotes, with the exception of trypanosomes that lack the entire complex. Here, I show that trypanosomes have evolved to use an ApaH-like phosphatase instead of a nudix domain protein as their major decapping enzyme. This work closes an important gap in the knowledge of trypanosome mRNA metabolism. Moreover, this is the first reported function of an ApaH-like phosphatase, a bacterial derived class of enzymes that are widespread throughout eukaryotes.

## Introduction

One hallmark of eukaryotic mRNAs is the mRNA cap, a 7-methyl-guanosine bound 5’-5’ to the mRNA’s 5’ end by a triphosphate bridge. Together with the poly(A) tail that is connected to the cap via the poly(A) binding protein and the eIF4F complex, the cap mediates mRNA circularisation and contributes to mRNA stabilisation. For mRNA degradation, the circular structure is resolved by the removal of the poly(A) tail by the deadenylase of the Caf1/Ccr4/Not complex. Deadenylated mRNAs are then targets for one of two alternative decay pathways. mRNA can be degraded 3’-5’ by the exosome followed by the hydrolysis of the remaining capped di- or oligo-nucleotides by the pyrophosphatase DcpS. Alternatively, mRNA is decapped by the Dcp1/Dcp2 complex, followed by 5’-3’ exonucleolytic decay by the major cytoplasmic exoribonuclease XRN1. In many eukaryotes, including yeast and trypanosomes, 5’-3’ decay is the dominant mRNA decay pathway. Enzymes of this decay pathway localise to RNA granules, cytoplasmic RNA protein aggregates of largely unknown function. Best studied are P-bodies, which are constitutively present and contain the mRNA decay machinery and stress granules, which are induced by exposure to cellular stress and contain translation initiation factors [1,2]. RNA granules are present in all eukaryotes, including trypanosomes [3].

The nudix hydrolase Dcp2 is the catalytic component of the decapping complex [4–7] Dcp2 cleaves the mRNA cap between the α and β phosphate, releasing m^7^GDP and 5’-end monophosphate RNA [4,7]. Dcp1 binds to Dcp2 and acts as an activator [8,9]. The activity of the Dcp1/Dcp2 complex is further increased by several decapping enhancers; the ones conserved from yeast to human are Edc3, Pat1, Dhh1, Scd6 and the Lsm1-7 complex [10]. Edc3 and Pat1 bind and stimulate Dcp2 directly [11,12]. Pat1 binds and recruits the Lsm1-7 complex which mediates the selective binding to deadenylated mRNA substrates [13]. The effects of the DEAD-box RNA helicase Dhh1 and the Lsm domain protein Scd6 on decapping are probably only indirectly: both repress translation which increases the amount of decay-competent mRNA substrates [14–16]. At least in mammals, two additional nudix domain proteins are involved in decapping a subset of mRNAs [10].

To date, mRNA decapping by nudix domain proteins, in particular by Dcp2, is the only known mechanism of mRNA cap removal in eukaryotes. The enzyme is widespread throughout the eukaryotic kingdom: it was described in yeast [17], mammals [7] and plants [18], but also in several deep-branching protozoa, for example *Entamoeba histolytica* [19] and *Giardia lamblia* [20]. One marked exception are Kinetoplastida, a heterogenic group of flagellated protozoa that include some prominent human pathogens such as *Trypanosoma brucei*, *Trypanosoma cruzi* and *Leishmania*. Kinetoplastida have neither homologues to Dcp1 and Dcp2 nor to the major decapping enhancers Pat1 or Edc3 [21] and they do not possess a cytoplasmic Lsm1-7 complex [22,23]. Only the translational repressors Dhh1 [24–26] and Scd6 [27,28] are conserved. Despite of the absence of the decapping complex, the release of m^7^GDP from mRNAs is detectable *in vitro* [29]: trypanosomes must have evolved a mechanism for mRNA decapping, that is different to the one of other eukaryotes. One reason for the trypanosome’s need of a different decapping mechanism could be the unusual mRNA cap that in Kinetoplastida is transferred from the spliced leader RNA to all polycistronically transcribed mRNAs in a trans-splicing reaction [30]. The cap is of the heavily methylated type 4: the first four transcribed nucleotides (AACU) have ribose 2’-O methylations and there are additional base methylations on the first (m^6^_2_A) and fourth (m^3^U) position [31,32]. Importantly, the differences in mRNA decay between Kinetoplastids and other eukaryotes are restricted to the decapping complex. Trypanosomes have conserved homologues to all other mRNA decay components, including the exosome [33], the Xrn1 homologue XRNA [34,35] and the CAF1/NOT complex [36,37].

We have recently purified starvation stress granules from trypanosomes [38]. One of the newly identified granule proteins, here renamed ALPH1, is a strong candidate for the long-wanted trypanosome decapping enzyme, based on its predicted substrate specificities and on its exclusive co-localisation with XRNA to a special granule at the posterior pole of the cell. RNAi experiments confirm that TbALPH1 has all features required of an mRNA decapping enzyme: ALPH1 depletion is lethal and results in a massive, global increase in mRNAs, that are deadenylated but have not yet started degradation. *In vitro* experiments confirm that ALPH1 can sensitise a capped RNA to Xrn1 in the presence of an RNA 5’ polyphosphatase.

## Results

### Discovery and sequence analysis of TbALPH1

The protein with the Gene ID number Tb927.6.640 was identified both in stress granules [38] and within the mRNA-bound interactome [39]. Interestingly, the protein did not only localise to stress granules, but also to the posterior pole granule [38], a localisation that was so far only observed for the 5’-3’ exoribonuclease XRNA [40]. This observation indicates that Tb927.6.640 might belong to the same pathway as XRNA and perhaps is the long-wanted decapping enzyme.

**Fig 1:**
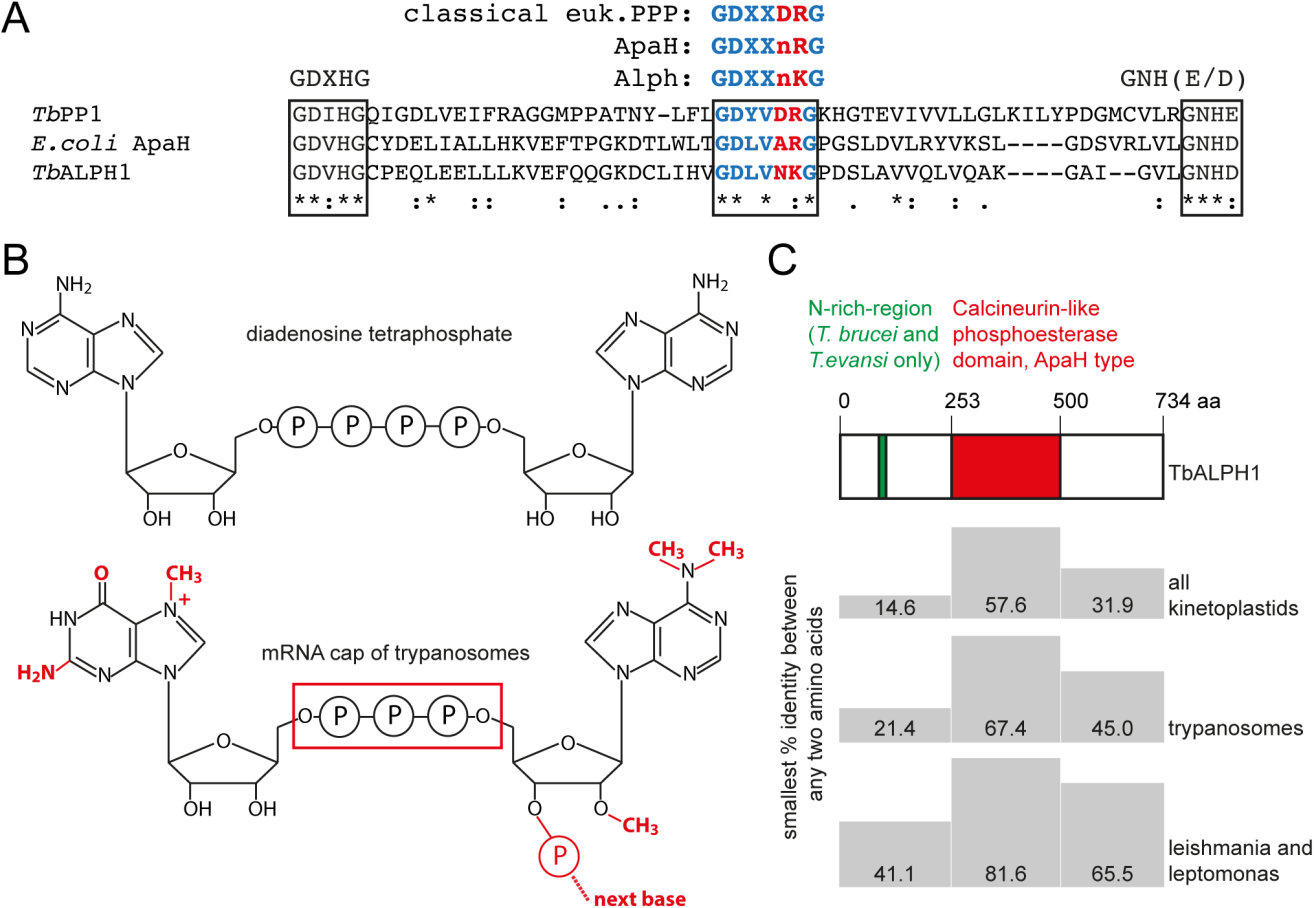
*T*. *brucei* ALPH1. (A) Alignment of the signature motifs of a classical eukaryotic PPP (TbPP1, Tb927.4.3560) and the atypical PPPs ApaH of *E*. *coli* and ALPH1 of *T*. *brucei*. The three catalytic signature motifs of PPPs are framed. The two changes in the GDXXDRG motif that are characteristic for ApaH and Alph are highlighted in red. In Alph’s and ApaH, the second aspartate is replaced by a neutral amino acid (n). In most Alphs, but not in ApaH or other PPPs, arginine, a coordinator of phosphate [72], is replaced by lysine [41]. (B) Structure of diadenosine tetraphosphate (top) and of the mRNA cap of trypanosomes (bottom). The differences are marked in red. (C) Domain structure of ALPH1 and conservation between the different kinetoplastida orthologues. All available kinetoplastid orthologues to *Tb*ALPH1, (13 sequences of trypanosomes, 1 of *Leptomonas* and 6 of *Leishmania* strains) were aligned by ClustalW using default parameters. The minimal % identity between any two sequences is indicated for the three different ALPH domains. The sequences of trypanosomes and *Leishmania*/*Leptomonas* were also analysed separately.

Tb927.6.640 is annotated as a kinetoplastid-specific phospho-protein phosphatase (PPP) in the TriTryp database [21]. A closer analysis of the sequence reveals two changes in the conserved PPP signature motif GDXXDRG: the second aspartate is replaced by asparagine and arginine is replaced by lysine (Fig 1A). These changes are characteristic for an ApaH-like phosphatase (Alph), a subgroup of the PPP family that is closely related to the bacterial enzyme ApaH [41,42]. I will refer to Tb927.6.640 as TbALPH1. There are two further ApaH-like phosphatases in the *T*. *brucei* genome: Tb927.4.4330 (TbALPH2) and Tb927.8.8040 (TbALPH3) (S1 Fig), but neither was identified as stress granule component [38] or as involved in mRNA binding [39]. The substrates of eukaryotic ApaH-like phosphatases are unknown, even though these proteins are widespread throughout the eukaryotic kingdom [41]. The substrate of the bacterial ApaH protein, however, is diadenosine tetraphosphate [43,44], a molecule that resembles an eukaryotic mRNA cap (Fig 1B).

TbALPH1 has 734 amino acids and is almost equally split into a C-terminal domain, the catalytic domain and an N-terminal domain (Fig 1C). ALPH1 of *T*. *brucei* and *T*. *evansi* also has an asparagine-rich region within the N-terminal domain. The protein appears to be unique to Kinetoplastida; there are similarities to both Alph’s of other eukaryotes and to bacterial ApaH proteins, but these are restricted to the catalytic domain. When all available ALPH1 sequences of the Kinetoplastida are aligned, there are 14.6%, 57.6% and 31.9% minimal identity between any two sequences in the C-terminal part, catalytic domain and N-terminal part of the protein, respectively (Fig 1C). Thus, the catalytic domain is best conserved, followed by the C-terminal domain and a rather poorly conserved N-terminal domain. When the ALPH1 sequences of Trypanosomes and *Leishmania*/*Leptomonas* were aligned separately, the percentages of minimal identities increased, but the N-terminal domain remained the least- and the catalytic domain the best-conserved domain (Fig 1C).

### ALPH1 RNAi depletion causes growth arrest and increase in mRNA levels

**Fig 2:**
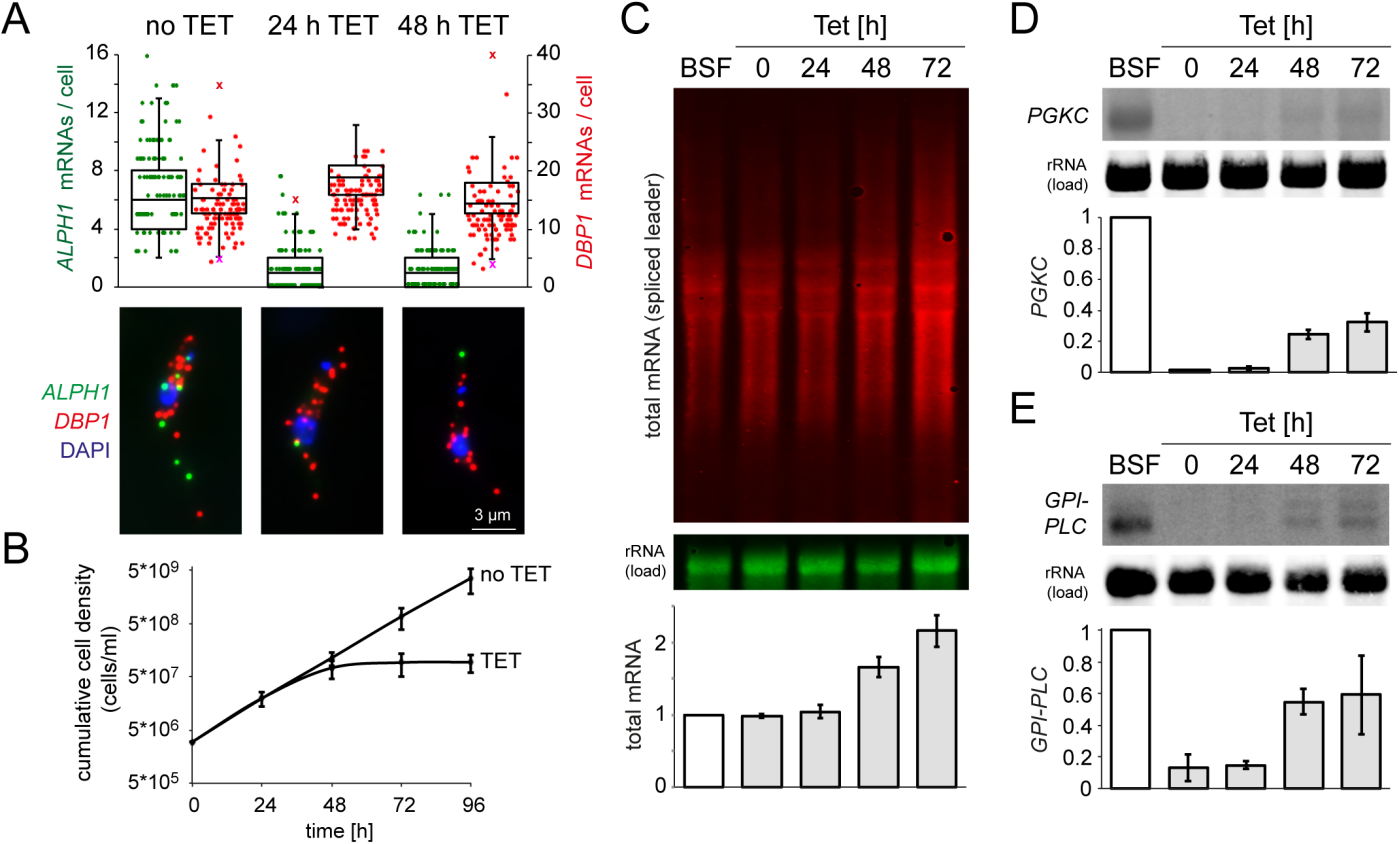
RNAi depletion of ALPH1 causes growth arrest and increase in mRNA levels. RNAi depletion of TbALPH1 was induced by tetracycline (TET). Three independent clonal cell lines were analysed over a time-course of RNAi induction. (A) Reduction in the number of *ALPH1* mRNA molecules. *ALPH1* mRNA and *DBP1* mRNA (control) were visualized by dual colour single molecule mRNA FISH (Affymetrix), using green (*ALPH1*) and red (*DBP1*) fluorescent Affymetrix probe sets. The number of mRNA molecules per cell was quantified after 0, 24 and 48 hours of ALPH1 RNAi induction. Data are presented as box plot (waist is median; box is IQR; whiskers are ±1.5 IQR; only the smallest and largest outliers are shown; *n* = 100 for each time-point); the number of mRNAs from the individual cells is also presented as green (*ALPH1*) or red (*DBP1*) dots. One representative cell for each time-point is shown. The data are from one RNAi clone; data of a second clone are shown in S12 Fig. (B) Growth arrest. Growth was measured over a time-course of ALPH1 RNAi induction (±TET). Averages of the three clonal cell lines are shown; error bars indicate standard deviations between the three cell lines. (C-E) Increase in mRNAs. Total RNA was isolated over a time-course of ALPH1 RNAi and as a control from bloodstream form trypanosomes (BSF) and analysed by northern blots. The blots were probed for total mRNA with an oligo antisense to the miniexon of the spliced leader RNA (C), for *PGKC* (D) and for *GPI-PLC* (E). mRNA abundances were quantified by Odyssey (total mRNA) or phosphorimager (*PGKC*, *GPI-PLC*). rRNA was used as a loading control and all samples were calibrated to the amount of BSF RNA (= 1). Average values of the three clones are shown, standard deviations are presented as error bars. For each probe, one representative northern blot is shown. Note that the three red bands in C) are not rRNA bands, but are caused by a squeezing of the mRNAs due to the very abundant rRNA.

If ALPH1 is involved in decapping of bulk mRNAs, its depletion should be lethal and cause stabilisation of total mRNAs. RNAi depletion of TbALPH1 was performed with the tetracycline inducible system described in [45]. Three populations of cells of clonal origin were analysed. The reduction in Tb*ALPH1* mRNA was controlled by Affymetrix single molecule FISH with a green fluorescent probe antisense to *ALPH1* and a red fluorescent probe antisense to a control mRNA (*DBP1*). There was a clear reduction in the number of *ALPH1* mRNAs (from 6 to 1 molecule per cell), but not in the number of *DBP1* mRNAs within 24 hours of tetracycline induction in all three clones, indicating that the RNAi was successful (Fig 2A). Next, growth was monitored over a time-course of RNAi induction. Cells stopped growth within 48 and 72 hours of RNAi induction (Fig 2B), indicating that TbALPH1 is an essential protein.

The effects of ALPH1 RNAi depletion on mRNA levels were examined. Total RNA was isolated over a time-course of ALPH1 RNAi induction and analysed by quantitative northern blots (Fig 2C–2E). The blots were probed for total mRNAs with an oligo antisense to the miniexon sequence that is trans-spliced to all trypanosome mRNAs, and for two individual mRNAs. The two individual mRNAs, GPI-phospholipase C (*GPI-PLC*) and phosphoglycerate kinase C (*PGKC*) were chosen because they are unstable in the procyclic life cycle stage, in which the experiments were performed [46–49]; unstable mRNAs show a larger increase in steady state levels at inhibition of RNA decay. Total RNA from bloodstream form (BSF) trypanosomes served as a control. RNAi depletion of ALPH1 caused a significant increase in mRNA levels for all mRNAs analysed. Total mRNA levels increased 1.7/2.2 fold after 48/72 hours of ALPH1 RNAi induction. For the two developmentally regulated mRNAs the increase was higher: *GPI-PLC* mRNA increased 4.2/4-6 fold and *PGKC* mRNA increased 16/22 fold after 48/72 hours of RNAi induction.

The data suggest that ALPH1 has an essential role in trypanosome mRNA metabolism. The RNAi phenotype of ALPH1 is very similar to the phenotype observed after RNAi depletion of XRNA: cells stop growth [50], total mRNA levels increase about 2-fold (S2 Fig) and there is a particular pronounced effect on unstable, developmentally regulated mRNAs [35,51].

### The mRNAs that accumulate after ALPH1 RNAi depletion are deadenylated

If ALPH1 is the decapping enzyme, it should act downstream of mRNA deadenylation. Thus, the mRNAs that accumulate after ALPH1 depletion should have shorter poly(A) tails. Such a decrease in poly(A) tail length was observed after depletion of several yeast enzymes acting downstream of deadenylation, including Dcp2 [17], Xrn1 [52], Dcp1 [53] and Lsm1 [54].

In trypanosomes, the histone H4 mRNA is frequently used to report changes in poly(A) tail lengths [34,36]. The genes encoding histone H4 are organised as ten tandem repeats, producing mRNAs with an average size of 520 nucleotides (range 469–589), excluding the poly(A) tail. The small size of this mRNA allows the visualisation of changes in poly(A) tail lengths as band shifts on a northern blot, because the poly(A) tail occupies a large fraction of the total mRNA size [34,36].

**Fig 3:**
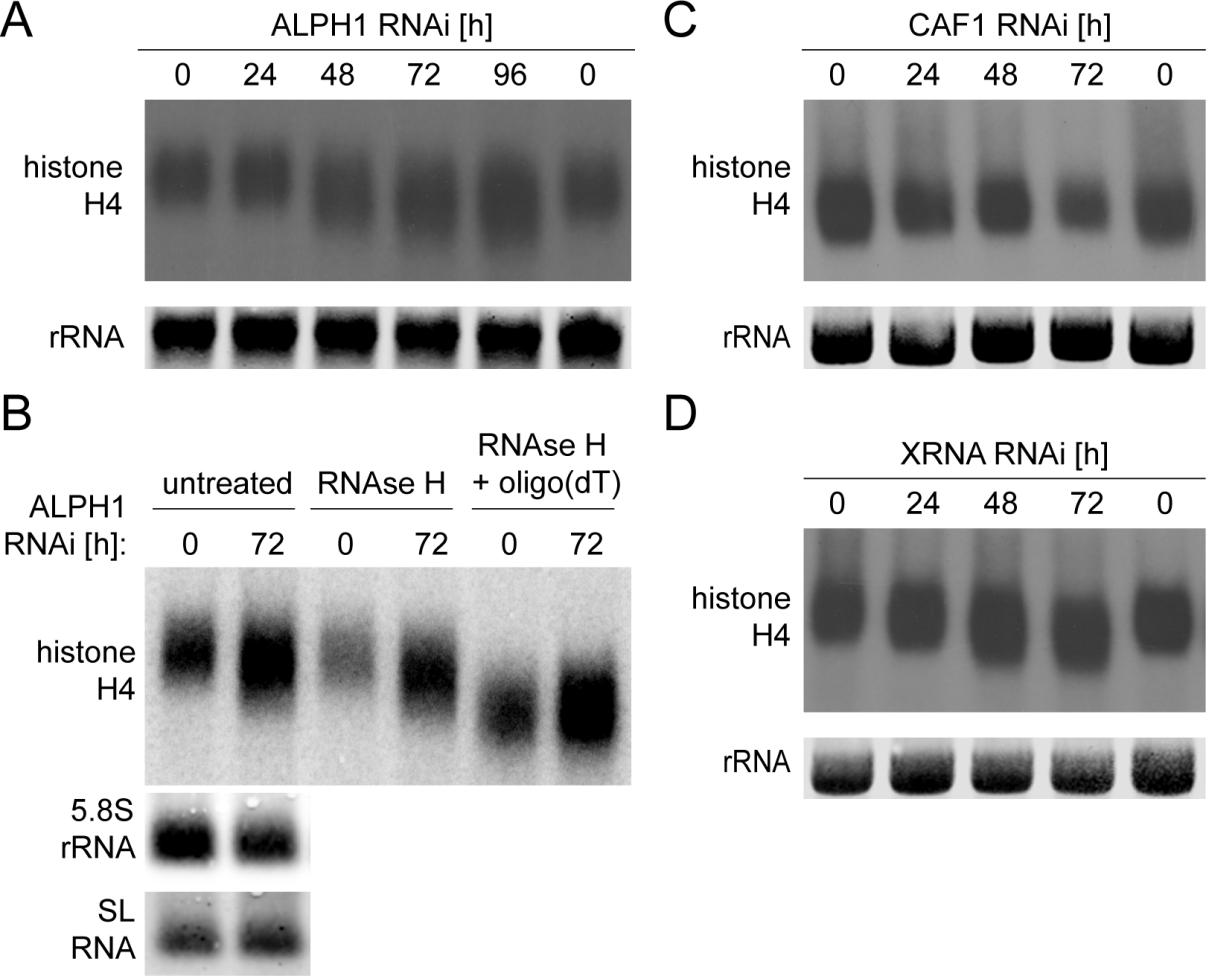
mRNAs that accumulate after ALPH1 RNAi are deadenylated. Northern blots were probed for *histone H4*, a very small mRNA that allows to visualise changes in poly(A) tail length by band shifts. (A) RNA was isolated over a time-course of ALPH1 RNAi depletion. Representative data from one of three RNAi clones are shown. (B) RNA isolated after 0 or 72 hours of ALPH1 RNAi depletion was treated with RNAse H in the absence or presence of oligo dT. Samples not treated with RNAse H served as control. The blot was re-probed for two small RNAs that have no poly(A) tail (5.8S rRNA and SL RNA) to demonstrate that the band-shift is specific to polyadenylated RNAs. (C) RNA was isolated over a time-course of CAF1 RNAi depletion. Representative data from one of three RNAi clones are shown. (D) RNA was isolated over a time-course of XRNA RNAi depletion. Representative data from one of two RNAi clones are shown.

When a northern blot with RNA harvested over a time-course of ALPH1 RNAi was probed for histone H4, there was a clear decrease in mRNA size starting at 48 hours of tetracycline induction (Fig 3A). Two experiments served to control that the band-shifted mRNA is indeed deadenylated (Fig 3B): First, when the RNA samples were treated with RNAse H in the presence of oligo (dT), the band shift was lost. Second, no band shift is detectable in RNAs without a poly(A) tail, namely the 5.8S rRNA or the SL RNA, even though both are significantly smaller than *histone H4*. As a negative control, I performed RNAi specific to the deadenylase CAF1 (S3 Fig). There was a mild increase in histone H4 mRNA size (Fig 3C), as previously reported [36]. As a positive control, I performed RNAi specific to XRNA [50]. I observed a decrease in histone H4 mRNA size (Fig 3D), as previously reported [34], similar to the effect seen after ALPH1 RNAi. The data are consistent with ALPH1 acting downstream of the mRNA deadenylase CAF1 on deadenylated mRNA substrates.

### ALPH1 RNAi depletion causes an increase in intact mRNA molecules, but no increase in decay intermediates

So far, the RNAi phenotypes of XRNA and ALPH1 are identical. Both are essential proteins and RNAi depletion causes an accumulation of deadenylated mRNAs, suggesting that both enzymes act downstream of deadenylation. However, if ALPH1 is the decapping enzyme, it should be involved in the initiation of mRNA decay, while XRNA is involved in the actual mRNA degradation process.

**Fig 4:**
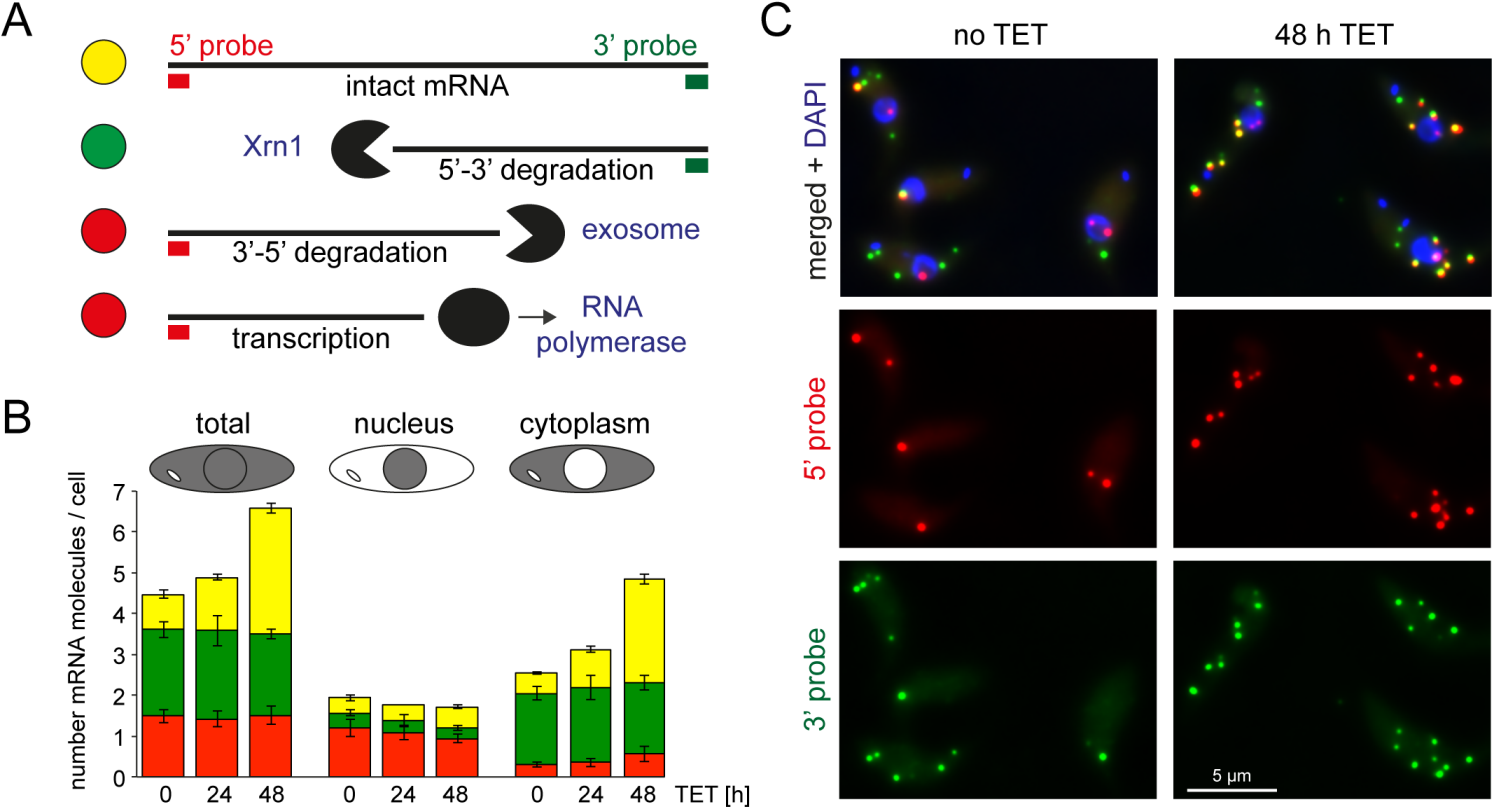
RNAi depletion of ALPH1 causes an increase in intact mRNA molecules, but no increase in 5’-3’ decay intermediates. (A) Experimental design: The endogenous very long and short-lived mRNA Tb427.01.1740 was used as a reporter for mRNA metabolism [55]. Simultaneous probing of the extreme 5’ and 3’ ends with red and green fluorescent single mRNA FISH probes results in yellow, green or red fluorescent mRNA molecules, corresponding to intact mRNAs, mRNAs in 5’-3’ decay and mRNAs in transcription or 3’-5’ decay, respectively. (B) The numbers of yellow, green and red fluorescent spots were quantified from cells after induction of ALPH1 RNAi (0, 24 or 48 hours TET) from at least 130 cells per time-point, for three RNAi clones. Average data are shown; error bars represent the standard deviations between the different RNAi clones. The numbers of the differently coloured spots per total cell are shown (left), but also the numbers of spots in the nucleus (middle) or in the cytoplasm (right). If the centre of a spot was overlapping with the DAPI stained nucleus on a Z-stack projection image, the spot was defined as nuclear, otherwise as cytoplasmic. (C) Representative Z-stack projection images of untreated cells (no TET) and cells after 48 hours of ALPH1 RNAi (48 h TET).

I have recently developed a method that enables to distinguish between these two enzyme functions [55]. Briefly, an endogenous very long mRNA (Tb427.01.1740) is used as a reporter to visualise mRNA degradation intermediates. The extreme 5’ and 3’ ends of this mRNA are simultaneously probed with a red and green fluorescent single molecule mRNA FISH probe (Affymetrix), respectively. This way, intact mRNA molecules appear as yellow spots, mRNAs in 5’-3’ decay are green and mRNAs in 3’-5’ decay or in transcription appear as red spots (Fig 4A). Due to the large size and short half-life of the reporter mRNA, there are only 21% of intact mRNAs, but 48% are in 3’-5’ decay [55]. RNAi depletion of XRNA for 48 hours resulted in a 5.6 fold increase in yellow spots and a 4.3 fold increase in green spots [55] (S4 Fig). Thus, a reduced level of XRNA does not only cause an increase in intact mRNA molecules, but also an increase in 5’-3’ decay intermediates; the most likely explanation for this is that XRNA is not 100% processive over the extreme length of the reporter mRNA. Any early release of XRNA from its substrate thus causes an increase in 5’-3’ decay intermediates because the time of decay-reinitiation is increased due to the shortage in XRNA molecules. In contrast to the RNAi phenotype of XRNA, RNAi depletion of any enzyme involved in the initiation of mRNA decay should cause an increase in intact mRNA molecules (yellow spots) only.

To examine whether ALPH1 functions in mRNA decay initiation or mRNA degradation, cells were harvested over a time-course of ALPH1 RNAi and probed for the mRNA metabolism reporter Tb427.01.1740 [55]. The numbers of red, green and yellow spots were counted. There was a 1.5/3.5 fold increase in yellow spots (intact mRNA molecules) after 24/48 hours of RNAi induction, but no change in green or red spots (Fig 4B and 4C) (S5 Fig). The spots were further classified as either nuclear or cytoplasmic based on their co-localisation with the DAPI-stained nucleus on a Z-stack projection image. The increase in yellow spots was entirely due to an increase in cytoplasmic yellow spots (Fig 4B), as expected as a result of a decapping arrest.

These data now show a clear difference between the RNAi phenotypes of XRNA and ALPH1: depletion of XRNA RNAi results in an increase in both decay intermediates and intact mRNAs, while depletion of ALPH1 only causes an increase in intact mRNAs. The data suggest that ALPH1 acts upstream of XRNA in the initiation of mRNA decay, but downstream of deadenylation. The only known enzyme that fulfils these criteria is the decapping enzyme.

### ALPH1 co-localises exclusively with XRNA to the posterior pole

As part of a screen, we have previously shown that TbALPH1 localises to both the posterior pole of the cell as well as to starvation stress granules and P-bodies [38]. A localisation to the posterior pole was previously observed only for XRNA but for no other proteins involved in mRNA metabolism [40].

To investigate a possible co-localisation between TbALPH1 and TbXRNA, I expressed ALPH1 with a C-terminal eYFP tag from the endogenous locus, together with XRNA-mChFP. Importantly, a C-terminally tagged ALPH1 is fully functional, as cell lines entirely dependent on the fusion protein could be obtained by deleting the second ALPH1 allele and showed no reduction in growth as well as the correct localization of the fusion protein (S6 Fig). As a control, I also co-expressed TbALPH1-eYFP with an N-terminal mChFP fusion of the DEAD box RNA helicase DHH1, a P-body marker protein that is absent from the posterior pole [40].

**Fig 5:**
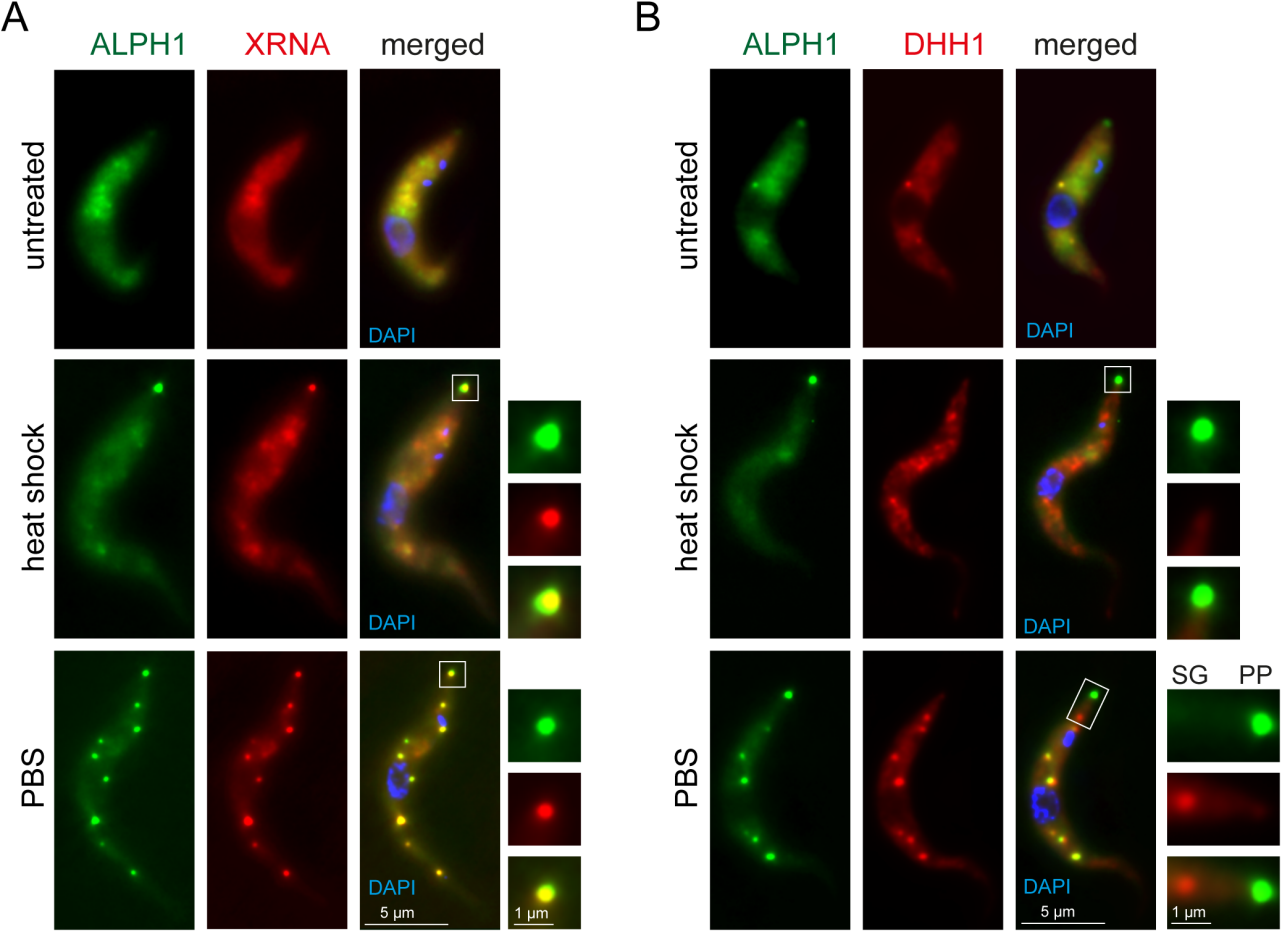
Only XRNA fully co-localises with ALPH1. (A) Cells co-expressing XRNA-mChFP and ALPH1-eYFP from endogenous loci were treated with heat shock or starvation (PBS). The posterior pole granule is shown enlarged. (B) Cells co-expressing mChFP-DHH1 and ALPH1-eYFP from endogenous loci were treated with heat shock or starvation (PBS). The posterior pole granule (PP) is shown enlarged; for the PBS-treated cells the enlargement also includes the most posterior stress granule (SG) to show the absence of ALPH1.

I observed full co-localisation between XRNA and ALPH1 to the posterior pole granule at heat shock as well as to both starvation stress granules and the posterior pole granule when cells were treated with PBS (Fig 5A and S7 Fig). In contrast, the co-localisation between ALPH1-eYFP and mChFP-DHH1 was only partial, as previously observed [38]. ALPH1 and DHH1 co-localised to P-bodies in untreated cells and to most starvation stress granules in starved cells (Fig 5B). DHH1 was absent from the posterior pole granule and ALPH1 was absent from some starvation stress granules, in particular from the ones localised closest to the posterior pole granule (Fig 5B and S8 and S9 Figs).

The exclusive co-localisation between ALPH1 and XRNA is further evidence for both proteins being members of the same pathway.

### Recombinant ALPH1ΔN sensitises the trypanosome SL RNA to yeast Xrn1 *in vitro*, but only after treatment with an RNA 5’ Polyphosphatase

The above data provide strong *in vivo* evidence for ALPH1 being the trypanosome decapping enzyme. To directly show the decapping activity *in vitro*, recombinant ALPH1 protein was purified from *E*. *coli*. The full length ALPH1 protein could not be expressed in a soluble form, and a truncated version lacking the N-terminal 221 amino acids (ALPH1**Δ**N) was therefore used. As a control, the same truncated version was expressed as an inactive mutant (Alph1**Δ**N*). For this, the highly conserved GDVHG motif involved in metal ion binding was mutated to GNVHG [56]. A Coomassie-stained gel with both purified proteins is shown in S10 Fig.

**Fig 6:**
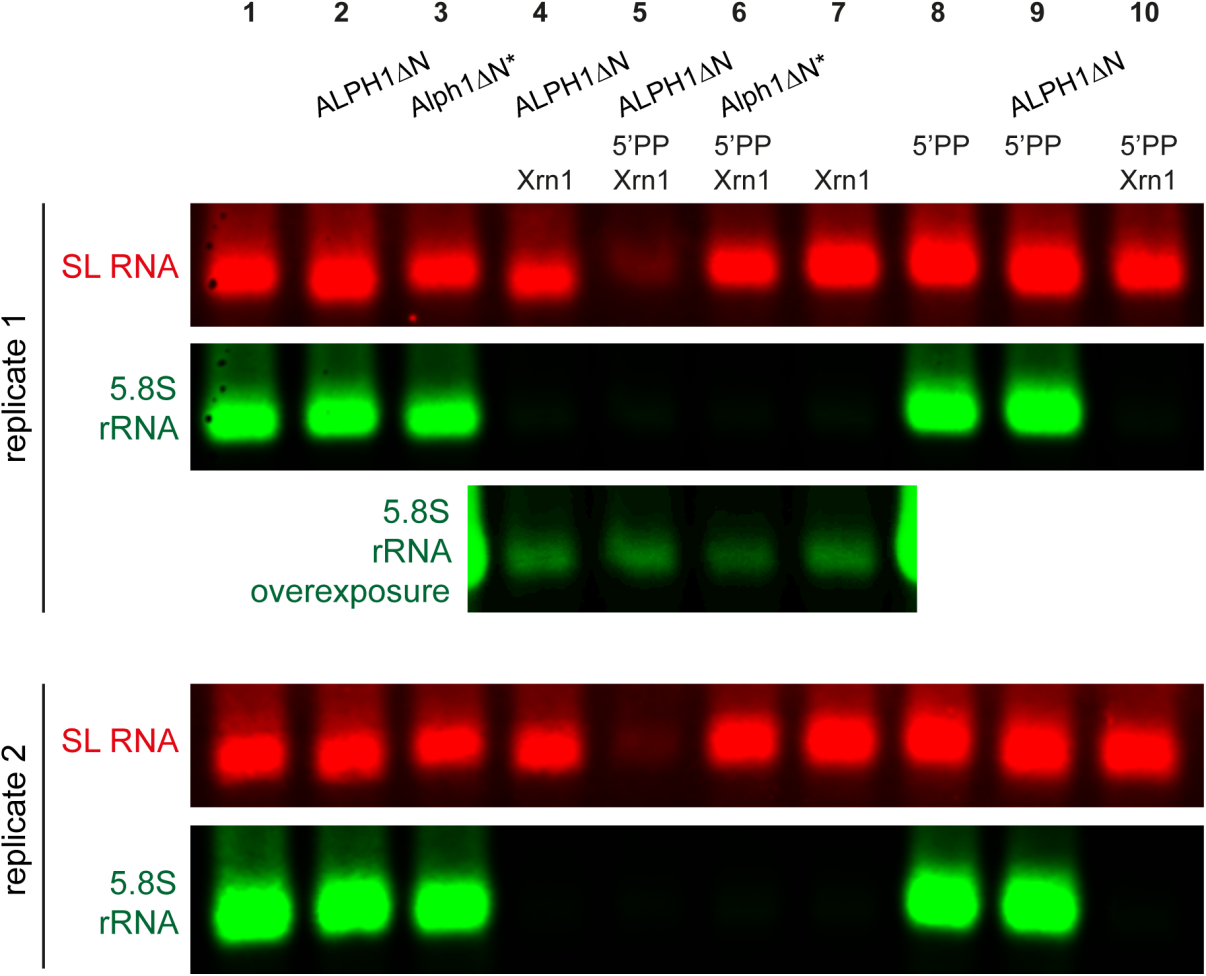
ALPH1ΔN has *in vitro* decapping activity, in the presence of an RNA 5’ polyphosphatase. Total trypanosome mRNA was treated with recombinant ALPH1**Δ**N or Alph1**Δ**N* as indicated, purified, treated with RNA 5’ polyphosphatase as indicated, purified, and finally treated with yeast Xrn1 as indicated. The RNA samples were analysed by northern blot probed for the SL RNA (red) and the 5.8S rRNA (green). For lane 4–7 of replicate 1, an overexposed blot of the 5.8S rRNA is shown to demonstrate equal loading. Data of two representative replicates are shown, out of a total of four replicates.

Next, the purified proteins were incubated with total trypanosome mRNA in the absence and presence of yeast Xrn1. If ALPH1 is the decapping enzyme, it should sensitise capped mRNAs to Xrn1. All RNA samples were analysed by northern blots probed for the capped SL RNA and, as a positive control, for the uncapped 5.8S rRNA (Fig 6). While the 5.8S rRNA disappeared in the presence of Xrn1, the ALPH1**Δ**N-treated SL RNA did not, indicating that ALPH1**Δ**N by itself does not produce RNA substrates for Xrn1 (Fig 6, lane 4). However, the addition of ALPH1**Δ**N, but not of the inactive Alph1**Δ**N* mutant, reproducibly caused a small band shift of the SL RNA that was absent in the similar-sized uncapped 5.8S rRNA (for example lane 3 and 4 in Fig 6). This indicated that ALPH1 targets the SL RNA, but without producing a substrate for Xrn1. One explanation is that ALPH1 does not produce a monophosphorylated mRNA, but a di- or triphosphorylated mRNA, which would be resistant to Xrn1 degradation. To test this, ALPH1-treated samples were treated with an RNA 5’ polyphosphatase (5’PP), which transforms tri- or diphosphorylated RNA into monophosphorylated RNA. When this phosphatase was included, subsequent Xrn1 treatment caused disappearance of the SL RNA band (Fig 6, lane 5). This was not observed with the catalytically inactive mutant Alph1**Δ**N* or with any other combinations of the enzymes (Fig 6, lanes 6–10). Thus, ALPH1 sensitises a capped RNA to yeast Xrn1 if an RNA 5’ polyphosphatase is included. This is consistent with ALPH1 being the decapping enzyme, producing a di- or triphosphorylated mRNA.

### No evidence for a function of TbDCP2 in decapping

Recently, one of the five trypanosome nudix domain proteins was renamed TbDCP2 because it has *in vitro* decapping activity [57]. The similarity to Dcp2 of other eukaryotes is low and the decapping activity is very poor, when the mRNA substrate had a type 4 cap [57]. Moreover, the protein was not purified with trypanosome RNA granules [38] and not identified as mRNA binding protein or protein involved in mRNA regulation in recent screens [39,58]. If TbDCP2 is responsible for the decapping of trypanosome mRNAs, its RNAi phenotype should resemble the RNAi phenotype of ALPH1. For three independent clonal cell lines, we found that RNAi depletion of TbDCP2 did not affect trypanosome growth, caused no increase in total mRNA levels and no change in the adenylation stage of the histone H4 mRNA (S11 Fig). Together, the data provide no evidence for TbDCP2 being the major trypanosome decapping enzyme and thus the functional orthologue to Dcp2.

## Discussion

I show that the trypanosome ApaH-like phosphatase ALPH1 has all characteristics of an mRNA decapping enzyme. The enzyme is essential, localises to P-bodies and its depletion causes the accumulation of deadenylated mRNA molecules that have not yet started degradation. Its exclusive co-localisation with the 5’-3’ exoribonuclease XRNA suggests that both enzymes are members of the same pathway. Importantly, total mRNA levels increase to the same extent upon TbALPH1 and XRNA RNAi (compare Fig 2C and S2 Fig), ruling out that TbALPH1 only targets a small sub-group of mRNA molecules. *In vitro* experiments show that ALPH1**Δ**N, but not an inactive mutant of ALPH1 can sensitise the capped SL RNA to yeast Xrn1, as long as an RNA 5’ polyphosphatase is included. In contrast, depletion of the nudix domain protein TbDCP2 that was previously suggested to be the decapping enzyme, did not affect growth, mRNA levels or mRNA adenylation state. This is consistent with its *in vitro* activity being very low when a mature type 4 cap was used as a substrate [57]. I propose that TbALPH1 is the major trypanosome decapping enzyme and thus the functional orthologue to DCP2 of other eukaryotes.

The *in vitro* decapping experiments provide evidence for the decapping mechanism of ALPH1 being fundamentally different from the DCP2-mediated decapping. The data are consistent with ALPH1 leaving either a tri- or a diphosphate at the 5’ end of the decapped mRNA, instead of a monophosphate. A diphosphate is more likely than a triphosphate, as the bacterial ApaH protein cleaves phosphoanhydrid bonds rather than phosphoester bonds. In previous *in vitro* decapping assays using cell extracts of the Trypanosomatid *Leptomonas*, three products were released from an artificial RNA substrate with a type 0 cap: m^7^GDP, m^7^GMP and m^7^GpppG [29]. The release of m^7^GMP was interpreted as the result of a cap scavenger activity [29], the degradation of the cap structure after 3’-5’ exosomal decay [59]. However, more recent data provide little evidence for a cytoplasmic function of the trypanosome exosome [50] and it is possible that the detected m^7^GMP is in fact the product of ALPH1. It remains an unsolved question, how the uncapped, poly-phosphorylated mRNA is further degraded. One possibility is that the trypanosome 5’-3’ exoribonuclease, unlike yeast Xrn1, accepts poly-phosphorylated mRNAs as a substrate. If this is not the case, the trypanosome decapping complex must contain an RNA phosphatase that produces monophosphorylated mRNA substrates for XRNA. Notably, all *in vitro* experiments were performed with an N-terminally truncated ALPH1 protein. Thus, there also remains the possibility that the N-terminus of ALPH1 affects the catalytic mechanism.

To date, all reported decapping enzymes that act on intact mRNA caps are nudix domain proteins. This includes DCP2, long believed to be the only eukaryotic decapping enzyme, as well as the more recently discovered enzymes Nudt3 and Nudt16 that are responsible for decapping a subset of mRNAs in mammals [10,60–63]. Even in bacteria, mRNA decay is initiated by the nudix hydrolase RppH, which cleaves pyrophosphate from the mRNA’s 5’ terminal triphosphate [64]. Homologues to DCP2 are readily detectable in representative organisms of all major eukaryotic super-groups defined by [65]: Opisthokonta (yeast, human), Amoebozoa (*Dictyostelium*), Archaeplastida (*Arabidopsis*), SAR (*Plasmodium*), CCTH (*Cryptophyta*, *Haptophyta*) and Excavata (*Naegleria*, *Trichomonas*). Loss of DCP2 is selectively observed in (at least) two subgroups of the Excavata, the Euglenozoa (*Kintoplastida* and *Euglena gracilis*) and Fornicata (*Giardia intestinalis* and *Spironucleus salmonicida*). Only the kinetoplastid *Perkinsela* has a DCP2 homologue; it may be the product of a lateral gene transfer event as it has closest homology to DCP2 of plants and *Perkinsela* lacks all other components of the decapping complex. Does the loss of DCP2 correlate with the gain of ALPH1? All *Kinetoplastida* and *Euglena* do have a homologue of TbALPH1. The ALPH1 homologue of *Euglena gracilis* constitutes almost solely of the catalytic domain, but is still more similar to TbALPH1 than to TbALPH2 or TbALPH3. In contrast, neither *Giardia intestinalis* nor *Spironucleus salmonicida* has a homologue to TbALPH1. Thus, ALPH1 has replaced DCP2 in *Euglenozoa*, but *Fornicata* must compensate the absence of DCP2 in a different way. Notably, there are many eukaryotes that have ApaH like phosphatases in addition to a DCP2 homologue; these include eukaryotes of all branches, with the marked exception of vertebrates, insects or land plants [41,66]. The homology to ALPH1 is restricted to the catalytic domain and whether these are decapping enzymes, acting in addition to DCP2, or have evolved a different function, remains to be investigated. To my knowledge, no function of an ApaH-like phosphatase has yet been reported.

The described changes in mRNA decapping of *Euglenozoa* are not restricted to the replacement of DCP2 by an ApaH like phosphatase: the genomes of Kinetoplastids and *Euglena gracilis* also lack readily identifiable homologues to the decapping enhancers DCP1, EDC3 and Pat1. This indicates that the mechanistic differences between the mRNA decapping reactions of *Euglenozoa* and all other eukaryotes are major. Why have *Euglenozoa* evolved a mechanism for mRNA decapping that is fundamentally distinct to mRNA decapping present in the rest of the eukaryotes? SL RNA based trans-splicing cannot be responsible, as this is also present in several other eukaryotes that do have a DCP2 homologue, for example Nematodes and Dinoflagellates. However, to the best of my knowledge, only SL RNAs of Kinetoplastids have been found to be of the heavily methylated type 4. A recent publication shows that mammalian mRNAs that have a N^6^,2′-O-dimethyladenosine as the first nucleotide following the m7G cap are resistant to decapping by Dcp2 [67]; the corresponding nucleotide of the trypanosome type 4 cap is with three methyl groups even heavier methylated (Fig 1B). Thus, the unusual mRNA cap structure of *Euglenozoa* may require an unusual decapping enzyme.

Uniquely, a fraction of TbALPH1 and XRNA co-localise to a special granule at the posterior pole of the cell that is devoid of any other RNA binding proteins. I never observed any accumulation of mRNA degradation intermediates at the posterior pole (Fig 4 and [55], indicating that it is not the place of mRNA decay. I propose that instead, the granule separates the decay enzymes ALPH1 and XRNA from their mRNA targets and thus flexibly regulates the amount of mRNA decay as needed by the cell. The fraction of XRNA at the posterior pole is highly variable and depends on the cellular state of mRNA metabolism [40] and I found the same for ALPH1 (S. Kramer, manuscript in preparation).

ApaH-like phosphatases are present in all branches of the eukaryotic kingdom. With ALPH1, I report the first function for a eukaryotic ApaH-like phosphatase. In the absence of DCP2, *Trypanosoma brucei*, and possibly all *Euglenoids* use ALPH1 as their major mRNA decapping enzyme. This is an example of convergent evolution. TbALPH1 is essential, has no homologue in vertebrates and there is a crystal structure available for the closely related TbALPH3 [68]. These are some requirements for the employment of ALPH1 as a drug target against human diseases caused by *Kinetoplastida*, such as Leishmaniasis or Trypanosomiasis.

## Material and methods

### Trypanosomes

*Trypanosoma brucei* Lister 427 procyclic cells were used for most experiments. All RNAi- and overexpression experiments were done in Lister 427 pSPR2.1 cells that express a TET repressor [45]. RNAi or overexpression transcripts are under the control of a TET operator and expression is thus inducible with tetracycline. Cells were cultured in SDM-79 [69] at 27°C and 5% CO_2_. Transgenic trypanosomes were generated using standard procedures [70]. All experiments used logarithmically growing trypanosomes. For starvation, one volume of cells was washed once in one volume PBS and cultured for 2 hours in one volume PBS. Heat shock was done for two hours at 41°C either in a thermoblock or waterbath.

### Plasmids and cloning

ALPH1 (SK335): ALPH1 open reading frame without stop codon in pJET1.2 (Thermo-Fisher Scientific) with N-terminal BspMI site to allow HindIII-compatible overhangs (sequence upstream of start codon: ACCTGCactAAGCTTCCGCCACC) and C-terminal BglII site (sequence downstream of last codon: GGTTCTagatctTGATCA) in frame to allow HindIII/BamHI compatible cloning into plasmids based on [71] or [45].

ALPH1 RNAi (SK309). The C-terminal 706 nts of the ALPH1 open reading frame were cloned head to head into p3666 [45].

XRNA RNAi (3862): as described in [50].

TbDCP2 RNAi (SK435). The C-terminal 698 nts of the DCP2 open reading frame were cloned head to head into p3666 [45].

CAF1 RNAi (SK431). The C-terminal 827 nts of the CAF1 open reading frame were cloned head to head into p3666 [45].

XRNA-mChFP, endogenous expression (SK346): The C-terminal 1107 nts of the XRNA ORF were cloned into p2705 [71]. The plasmid was linearized with NheI.

**Δ**ALPH1 (SK390): A blasticidine resistance cassette was flanked by the 560 nts upstream of the ALPH1 ORF and 607 nts downstream of the ALPH1 ORF to allow deletion of ALPH1 by homologous recombination.

ALPH1-eYFP-4Ty1, endogenous expression (SK169): The C-terminal 706 nts of ALPH1 were cloned into SK141 [38], which is p2710 [71] with 4-Ty1 tags downstream of the eYFP. The plasmid was linearized with SalI. In the text, this plasmid is often referred to as ALPH1-eYFP to avoid confusion, as the Ty1 tag is not needed for the experiments shown here.

ALPH1-eYFP, endogenous expression (SK348): The C-terminal 706 nts of ALPH1 were cloned into p2948, which is p2710 [71] with a hygromycin resistance instead of neomycin resistance. This plasmid was used instead of SK169 to allow co-expression of ALPH1-eYFP with XRNA-mChFP (SK346). The plasmid was linearized with MscI.

mChFP-DHH1, endogenous expression (p2845): as described in [40].

His-ALPH1**Δ**N for bacterial expression (SK391): The ALPH1 open reading frame lacking the N-terminal 663 nucleotides was cloned into a modified pET15b plasmid for expression in *E*. *coli*. This resulted in IPTG inducible expression of an ALPH1**Δ**N protein with an N-terminal extension of MGSSHHHHHHSSGLVPRGSHMELYFQEASAT.

His-Alph1**Δ**N* for bacterial expression (SK468): As SK391, but the GDVHG encoding sequence was mutated to the GNVHG encoding sequence (D:N; GAC:AAC).

### FISH experiments

Affmyetrix single molecule mRNA FISH was done as described previously [55] using the QuantiGene ViewRNA ISH Cell Assay (Affymetrix), protocol for glass slide format. Affymetrix probe sets were: ALPH1 (green, type 4): The N-terminal 1500 nts antisense to the ALPH1 ORF. DBP1 (red, type 1): the first 1260 nts antisense to the DBP1 ORF. CAF1 (red, type 1): antisense to the full ORF of CAF1. 5’ probe set (red, type 1, AF19): antisense to 288 nts upstream of the Tb427.01.1740 ORF followed by the first 812 nts of the ORF [55]. 3’ probe set (green, type 4): antisense to the last 656 nts of the Tb427.01.1740 ORF followed by the 444 nts downstream of the stop codon [55].

### Microscopy

Z-stack images (100 stacks at 100 nm distance) were taken with a custom build TILL Photonics iMic microscope equipped with a sensicam camera (PCO), deconvolved using Huygens Essential software (Scientific Volume Imaging B. V., Hilversum, The Netherlands). All images are presented as Z-projections (method sum sliced) produced by ImageJ software. eYFP was monitored with the FRET-CFP/YFP-B-000 filter, mCherry and type 1 Affymetrix probes with the ET-mCherry-Texas-Red filter, type 4 Affymetrix probes with the ET-GFP filter and DNA with the DAPI filter (Chroma Technology CORP, Bellows Falls, VT).

### Sequence analysis of ALPH1

The organisms used for the sequence analysis and alignments in Fig 1C were: *Trypanosoma brucei* TREU927, *Trypanosoma brucei* Lister strain 427, *Trypanosoma evansi* strain STIB 805, *Trypanosoma brucei gambiense* DAL 972, *Trypanosoma congolense* IL3000, *Trypanosoma vivax* Y486, *Trypanosoma cruzi marinkellei* strain B7, *Trypanosoma cruzi* Sylvio X10/1, *Trypanosoma cruzi* CL Brener Esmeraldo-like, *Trypanosoma cruzi* Dm28c, *Trypanosoma cruzi* CL Brener Non-Esmeraldo-like, *Trypanosoma rangeli* SC58, *Trypanosoma grayi* ANR4, *Leptomonas seymouri* ATCC 30220, *Leishmania tarentolae* Parrot-TarII, *Leishmania* sp. MAR LEM2494, *Leishmania aethiopica* L147, *Leishmania tropica* L590, *Leishmania mexicana* MHOM/GT/2001/U1103, *Leishmania gerbilli* strain LEM452. The ALPH1 sequence of *Trypanosoma grayi* ANR4 lacks the N-terminal domain, most likely due to a sequencing/database error; this organism was excluded from the N-terminal alignments.

### Quantitative northern blots

Northern blots were done as previously described [40]. mRNA was prepared with the miRNeasy kit instead of the RNeasy kit (both Qiagen), if the detection of small RNAs (SL-RNA or 5.8S rRNA) was aimed. 18S rRNA and 5.8S rRNA were detected with antisense oligos coupled to IRDye 800, namely 5′ -CCTTCGCTGTAGTTCGTCTTGGTGCGGTCTAAGAATTTC-3′ and 5’-ACTTTGCTGCGTTCTTCAACGAAATAGGAAGCCAAGTC-3’, respectively. Total mRNA and SL RNA were detected by an oligo antisense to the miniexon sequence (5′-CAATATAGTACAGAAACTGTTCTA ATAATAGCGTT-3′), coupled to IRDye 700.

Blot images were collected with the Odyssey Infrared Imaging System (LI-COR Biosciences, Lincoln, NE) and quantified with ImageJ. The *histone H4*, *GPI-PLC* and *PGKC* probes were radioactively labelled DNAs of the entire ORFs; for the *DCP2* probe the C-terminal 697 nts were used. Blot images were collected with a phosphorimager and analysed with ImageJ.

### Decapping assays

For the first step (decapping), each 100 μl reaction contained 10 μl NEB buffer 3 (100 mM NaCl, 50 mM Tris-HCl, 10mM MgCl_2_, 1mM DTT, pH 7.9), 2 μl Ribolock (Thermo Fisher Scientific), 20 μg total trypanosome RNA prepared with the miRNeasy kit (Qiagen) and if required 2 or 20 μl recombinant ALPH1**Δ**N or Alph1**Δ**N* (corresponding to about 800 ng protein), respectively. After one hour incubation at 37°C, 200 μl water was added to increase the volume and the RNA was cleaned by extraction with 300 μl phenol and subsequent ethanol precipitation in the presence of 2 μl RNA grade glycogen (Thermo Fisher Scientific). The pellet was resuspended in the amount of water needed for the 20 μl reaction of the second step (RNA 5’ polyphosphatase treatment). To each reaction, 2 μl RNA 5’ polyphosphatase buffer (Epicentre) and 1 μl Ribolock were added and if required, 1 μl RNA 5’ polyphosphatase (Epicentre). After one hour incubation at 37°C, 280 μl water was added and the RNA was cleared and precipitated as above, but without adding additional glycogen.

The pellet was resuspended in the amount of water needed for the 100 μl reaction of the third step (Xrn1 treatment). To each reaction, 10 μl NEB buffer 3 and 2 μl Ribolock were added and, if required, 2 μl recombinant yeast Xrn1 (NEB). The samples were incubated at 37° for one hour and the RNA was cleared and precipitated as above. The pellet was resuspended in 6 μl water and the entire sample was analysed by northern blot.

### Production of recombinant ALPH1

One litre of RosettaBlue competent bacteria cells (Novagen) carrying the plasmids SK391 or SK468 was started from an over night culture. At an OD of 0.8, protein expression was induced with 0.02% Isopropyl-β-D-thiogalactopyranosid (IPTG) for 2 hours. Cells were harvested (15 min, 10.000 g), resuspended in 40 ml buffer A (300 mM NaCl, 50 mM NaH_2_PO_4_, pH 8) and disrupted by ultrasonication (Sonifier B-12, Branson). The non-soluble material was pelleted by centrifugation (15 min, 20.000 g) and the soluble material (supernatant) was incubated with 3 ml Nickel-NTA agarose (Qiagen) equilibrated in buffer A for 60 min while rotating. The agarose was washed 3 times for 5 min in 50 ml buffer A and poured into a 2 ml Pierce centrifuge column. For washing, the column was loaded successively with 5 ml buffer A, 5 ml 10 mM imidazole and 5 ml 20 mM imidazole. Elution was done in two steps with 5 ml 100 mM imidazole and 5 ml 250 mM imidazole. Both eluates were rebuffered into PBS with PD10 columns (GE healthcare). The majority of the proteins eluted with 100 mM imidazole, but the 250 mM eluate contained less contaminants and was therefore used for the enzyme assays.

### RNAse H experiment

4 μg total RNA was incubated with or without 2 μl oligo (dT) (100 μM) in 16 μl volume at 65°C for 10 minutes. After slowly cooling down to room temperature, 2 μl RNAse H buffer, 1 μl Ribolock (ThermoScientific) and 1 μl RNAse H (NEB) or water (control) was added. The reactions were incubated at 37°C for one hour. RNA was purified by phenol-extraction followed by ethanol precipitation in the presence of 1 μl glycogen and 1 μl tRNA (25 mg/ml). RNA pellets were resuspended in 6 μl water for northern blot analysis.

## Supporting information

S1 Fig Alignment of the protein sequences of TbALPH1, TbALPH2 and TbALPH3. (PDF)

S2 Fig Increase in total mRNAs after tetracycline (TET) inducible RNAi depletion of XRNA. (PDF)

S3 Fig RNAi depletion of CAF1. Three independent clonal cell lines were analysed over a time-course of RNAi induction.
(PDF)

S4 Fig mRNA decay was monitored after 0 and 48 hours of XRNA RNAi (field view image). (PDF)

S5 Fig mRNA decay was monitored after 0 and 48 hours of ALPH1 RNAi (field view image). (PDF)

S6 Fig A cell line entirely dependent on Tbalph1-eYFP4Ty1 is viable. (PDF)

S7 Fig Starved cells co-expressing XRNA-mChFP and ALPH1-eYFP from endogenous loci (field view image). (PDF)

S8 Fig Starved cells co-expressing mChFP-DHH1 and ALPH1-eYFP from endogenous loci (field view image). (PDF)

S9 Fig Examples images of starved cells co-expressing ALPH1-eYFP and mChFP-DHH1 from the endogenous locus. (PDF)

S10 Fig Coomassie-stained gel of the purified proteins ALPH1ΔN and Alph1ΔN*. (PDF)

S11 Fig RNAi of TbDCP2 (Tb927.6.2670). (PDF)

S12 Fig RNAi depletion of ALPH1: The reduction in *ALPH1* RNA molecules was quantified by single molecule RNA FISH from a second clone. (PDF)
