# Supplementary material for: The ApaH-like phosphatase TbALPH1 is the major mRNA decapping enzyme of trypanosomes
Source: PLoS Pathog. 2017 Jun 19;13(6):e1006456. doi: 10.1371/journal.ppat.1006456 (PMC5491325; doi:10.1371/journal.ppat.1006456)
Supplement: S1 Fig — (PDF) [file ppat.1006456.s001.pdf]

Figure S1

```
                                                 classical euk.PPP: GDXXDRG
                                                              ApaH: GDXXnRG
                                             GDXHG            Alph: GDXXnKG
ALPH2 Tb927.4.4330         MKAQRYASVLTLDNLTGRLVVVGDIHGCLAQLQGLLRAVSFKQGSDTLVAVGDLVNKGP
ALPH3 Tb927.8.8040         MKVQGYANVVTLPNVTGRVIIVGDIHGCRAQLEDLLRAVSFKQGSDTLVAVGDLVNKGP
ALPH1 Tb927.6.640       ...DNVNAHKPIIRTVPPCDYRYIIVGDVHGCPEQLEELLLKVEFQQGKDCLIHVGDLVNKGP
                                   : *:     * ::***:***  **: **  *.*:**.* *: *********

                                            GNH(E/D)
ALPH2 Tb927.4.4330        DSFGVVRLLKRLGAHSVLGNHDVKLLKLVKKIREKGPLNERDTKSSLAPFALTVPVDVEV
ALPH3 Tb927.8.8040        DSFGVVRLLKRLGAYSVLGNHDAKLLKLVKKLGKKECLKGRDAKSSLAPLAQSIPTDVET
ALPH1 Tb927.6.640         DSLAVVQLVQAKGAIGVLGNHDFTLLNCIARVKGKAL-SQQEEVDPVMRLASTFPQGCED
                          **:.**:*::  ** .****** .**: : ::  *   . ::  . :  :* :.*   *

ALPH2 Tb927.4.4330        YLSQLPHIIRIPAHNVIVTHGGLHPQRPLDCQYIDEVTTLRNLIEKEQK-------AGGV
ALPH3 Tb927.8.8040        YLSQLPHIIRIPAHNVMVAHAGLHPQRPVDRQYEDEVTTMRNLIEKEQEA------TGGV
ALPH1 Tb927.6.640         YIRSLPHILRIPQYNVIVVHAGLNVENSLEKQNVHEIMHLRRLERYTESSNNNNNNCGGK
                          *: .****:*** :**:*.*.**. :. :: *  .*:  :*.* .  :.        **

ALPH2 Tb927.4.4330        T------LVATPETKDGGVPWASLWRGPETVVFGHDSRRGLQEQYRPLAIGLDSGCVYGG
ALPH3 Tb927.8.8040        T------LTATEETNDGGKPWASMWRGPETVVFGHDARRGLQEQYKPLAIGLDSRCVYGG
ALPH1 Tb927.6.640         QQRNEKQRLRAVVKGSRGQPWGELWTGPECVVFGHDARAGLQE--LPFAYGIDTGCVYGG
                                    :  . . * **..:* *** ******:* ****   *:* *:*: *****

ALPH2 Tb927.4.4330        RLSAAVFPGGHIVSVSGWRREAKL
ALPH3 Tb927.8.8040        RLSAAVFPGGCIISVPGWNGASAAA
ALPH1 Tb927.6.640         QLTAVVYGRD...
                          :*:*.*:
```

Figure S1: Alignment of the protein sequences of TbALPH1, TbALPH2 and TbALPH3
For Tb927.6.640 (ALPH1), only the catalytic domain is shown, Tb927.4.4330 (ALPH2) and Tb927.8.8040 (ALPH3) are shown in full length. The three signature motivs that are characteristic for all PPPs are framed. The GDXXGRD motif that is modified in a way that is characteristic for Alphs is shown in blue text, with the changes marked in red. The three Alphs are likely products of a recent gene duplication, as they are more similar to each other than to Alphs of non-Kinetoplastida. A structure is available for ALPH3 (PDB ID: 2QJC) (Almo et al., 2007).

Almo SC, Bonanno JB, Sauder JM, Emtage S, Dilorenzo TP, Malashkevich V, Wasserman SR, Swaminathan S, Eswaramoorthy S, Agarwal R, Kumaran D, Madegowda M, Ragumani S, Patskovsky Y, Alvarado J, Ramagopal UA, Faber-Barata J, Chance MR, Sali A, Fiser A, et al (2007) Structural genomics of protein phosphatases. J. Struct. Funct. Genomics 8: 121–140
