# Supplementary material for: The ApaH-like phosphatase TbALPH1 is the major mRNA decapping enzyme of trypanosomes
Source: PLoS Pathog. 2017 Jun 19;13(6):e1006456. doi: 10.1371/journal.ppat.1006456 (PMC5491325; doi:10.1371/journal.ppat.1006456)
Supplement: S2 Fig — (PDF) [file ppat.1006456.s002.pdf]

Figure S2

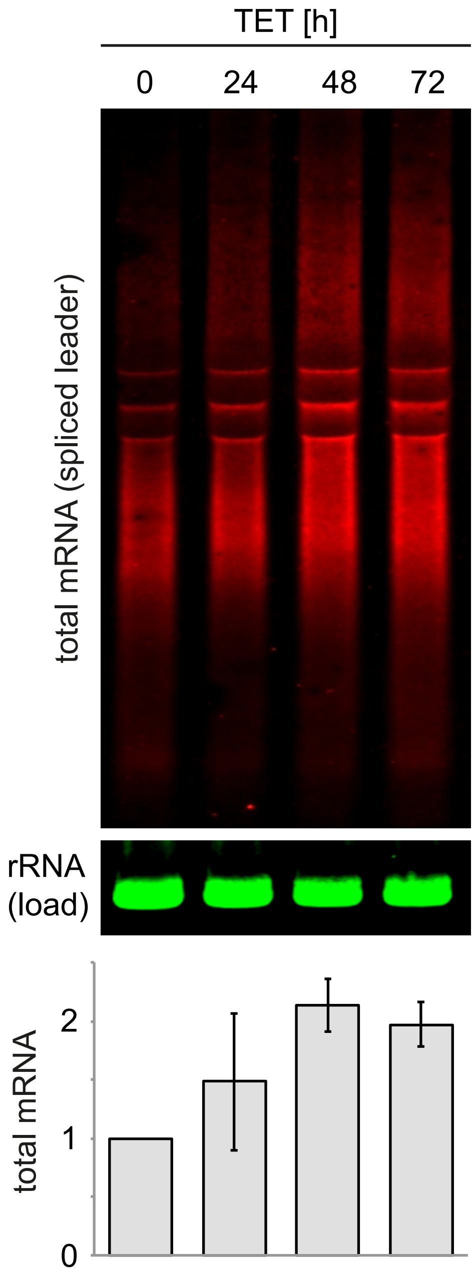

Figure S2: Increase in total mRNAs after tetracycline (TET) inducible RNAi depletion of XRNA. The data are averages from three independent clonal cell lines, standard deviations are indicated by error bars. One representative northern blot is shown.
