# Supplementary material for: The ApaH-like phosphatase TbALPH1 is the major mRNA decapping enzyme of trypanosomes
Source: PLoS Pathog. 2017 Jun 19;13(6):e1006456. doi: 10.1371/journal.ppat.1006456 (PMC5491325; doi:10.1371/journal.ppat.1006456)
Supplement: S3 Fig — Three independent clonal cell lines were analysed over a time-course of RNAi induction. (PDF) [file ppat.1006456.s003.pdf]

Figure S3

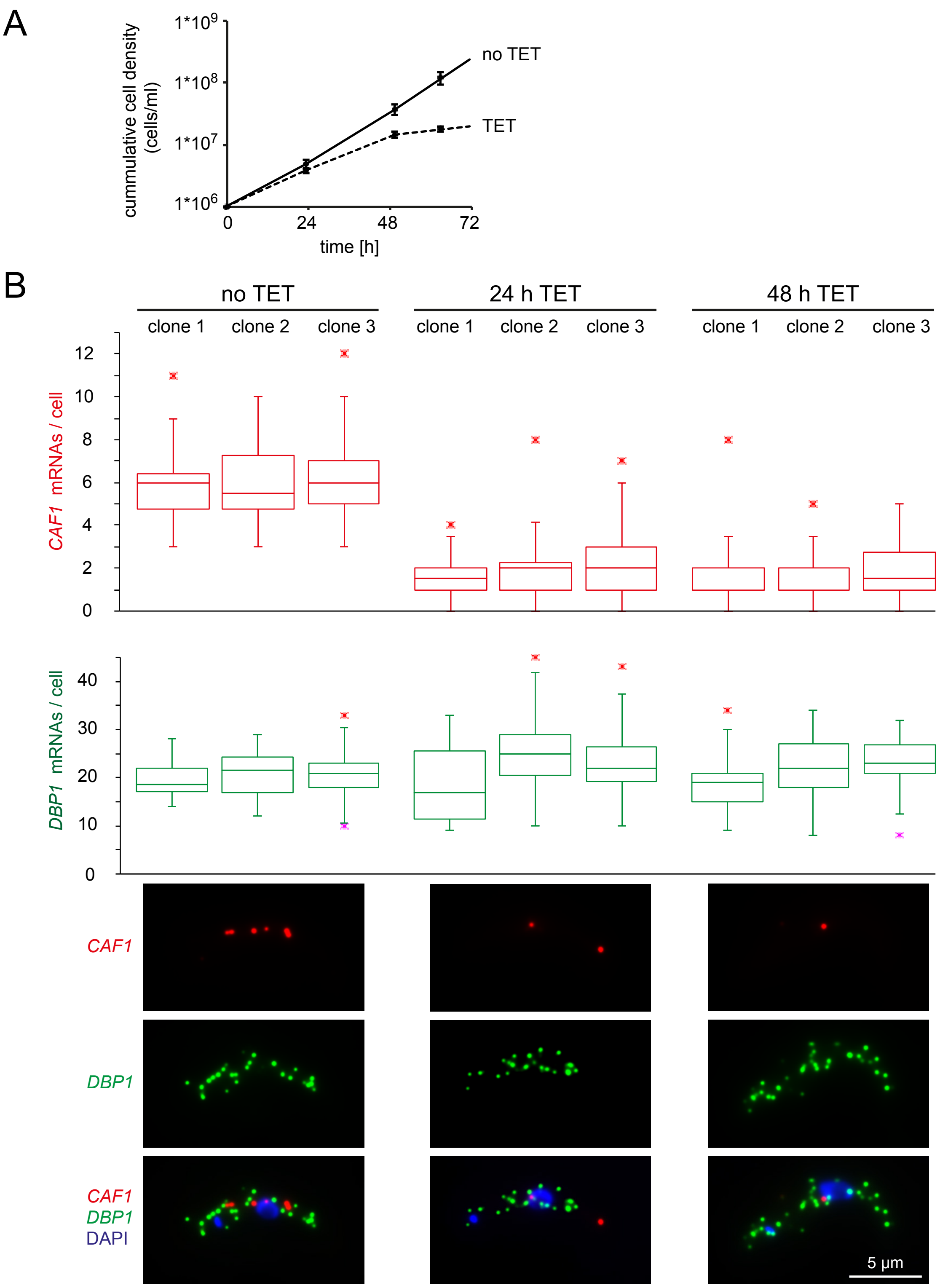

Figure S3: RNAi depletion of CAF1. Three independent clonal cell lines were analysed over a time-course of RNAi induction.

A) Growth curve. Growth was measured over a time-course of CAF1 RNAi induction (±TET). Averages of the three clonal cell lines are shown; error bars indicate standard deviations between the three cell lines.

B) Reduction in the number of *CAF1* mRNA molecules. *CAF1* mRNA and DBP1 mRNA (control) were visualized by dual colour, single molecule mRNA FISH (Affymetrix®), using red (CAF1) and green (DBP1) fluorescent Affymetrix probe sets. The number of mRNA molecules per cell was quantified after 0, 24 and 48 hours of CAF1 RNAi induction, for all three clonal cell lines. Data are presented as box plot (waist is median; box is IQR; whiskers are ±1.5 IQR; only the smallest and largest outliers are shown; n ≥14 for each time-point). One representative cell for each time-point is shown.
