# Supplementary figures and images for: The ApaH-like phosphatase TbALPH1 is the major mRNA decapping enzyme of trypanosomes
Source: PLoS Pathog. 2017 Jun 19;13(6):e1006456. doi: 10.1371/journal.ppat.1006456 (PMC5491325; doi:10.1371/journal.ppat.1006456)

Figure S4

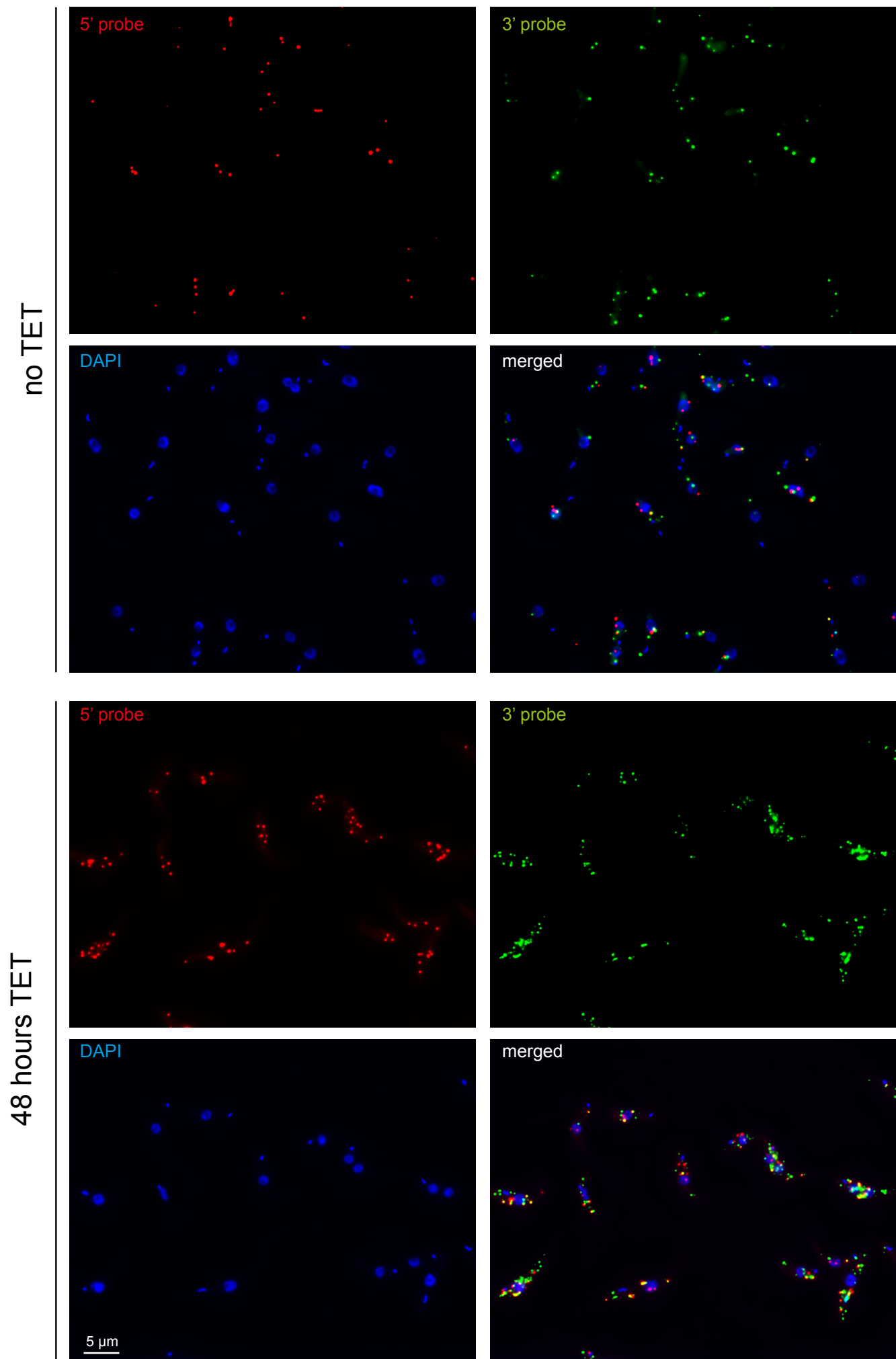

Figure S4: mRNA decay was monitored after 0 and 48 hours RNAi depletion of XRNA.

Supplement: S4 Fig — (PDF) [file ppat.1006456.s004.pdf]

Figure S5

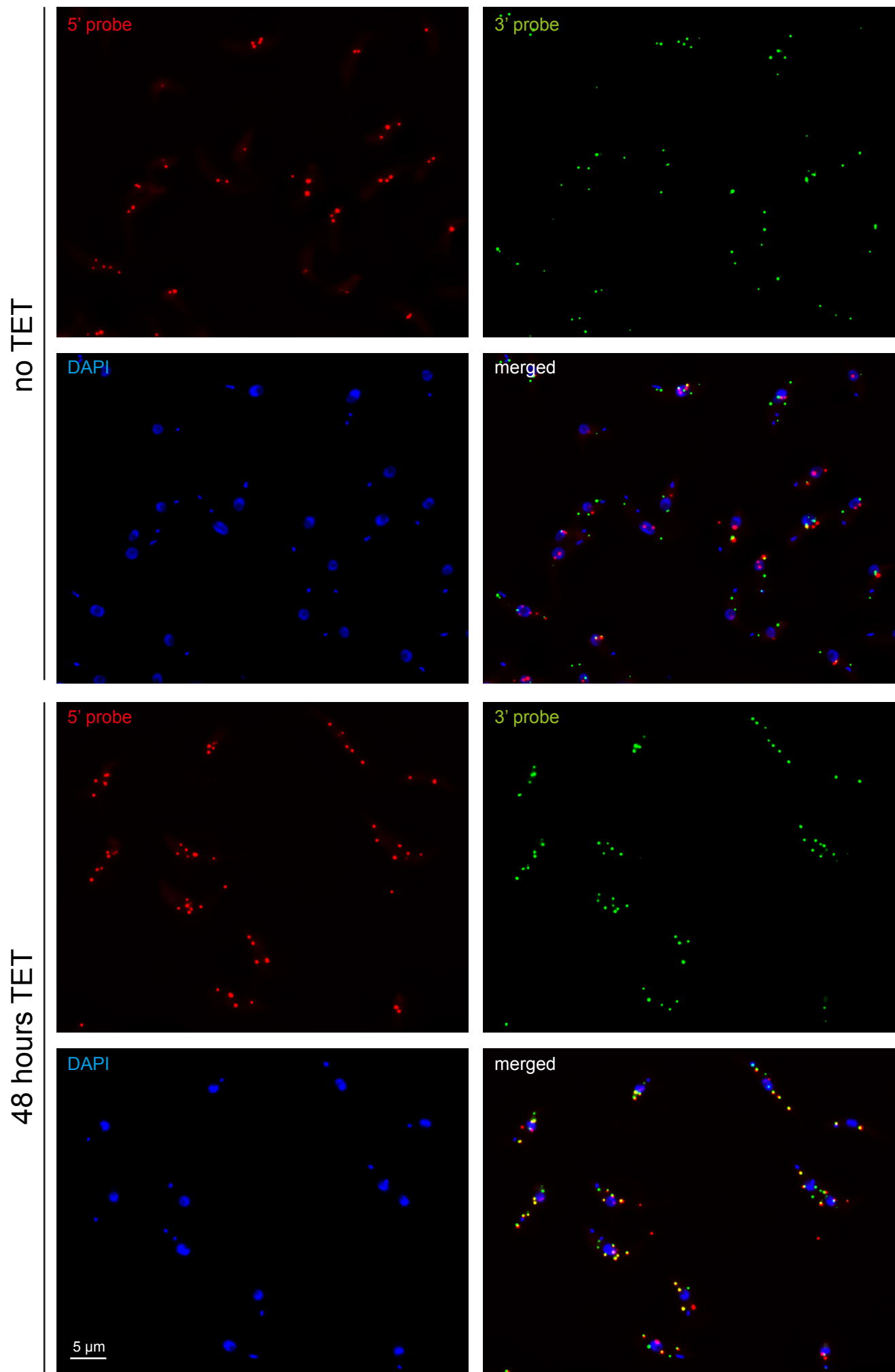

Figure S5: mRNA decay was monitored after 0 and 48 hours RNAi depletion of ALPH1.

Supplement: S5 Fig — (PDF) [file ppat.1006456.s005.pdf]

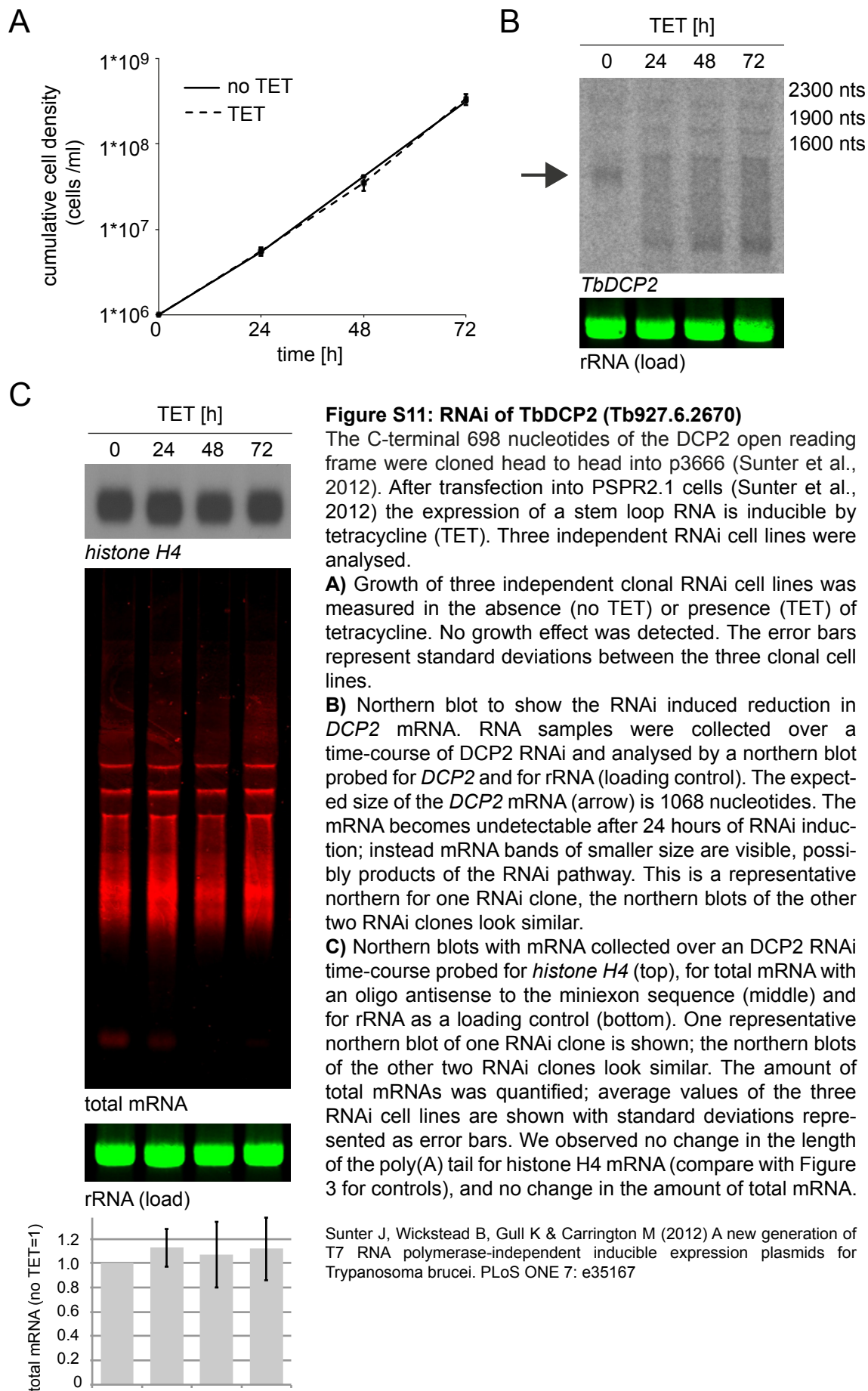

Supplement: S11 Fig — (PDF) [file ppat.1006456.s011.pdf]
