# Supplementary material for: The ApaH-like phosphatase TbALPH1 is the major mRNA decapping enzyme of trypanosomes
Source: PLoS Pathog. 2017 Jun 19;13(6):e1006456. doi: 10.1371/journal.ppat.1006456 (PMC5491325; doi:10.1371/journal.ppat.1006456)
Supplement: S6 Fig — (PDF) [file ppat.1006456.s006.pdf]

Figure S6

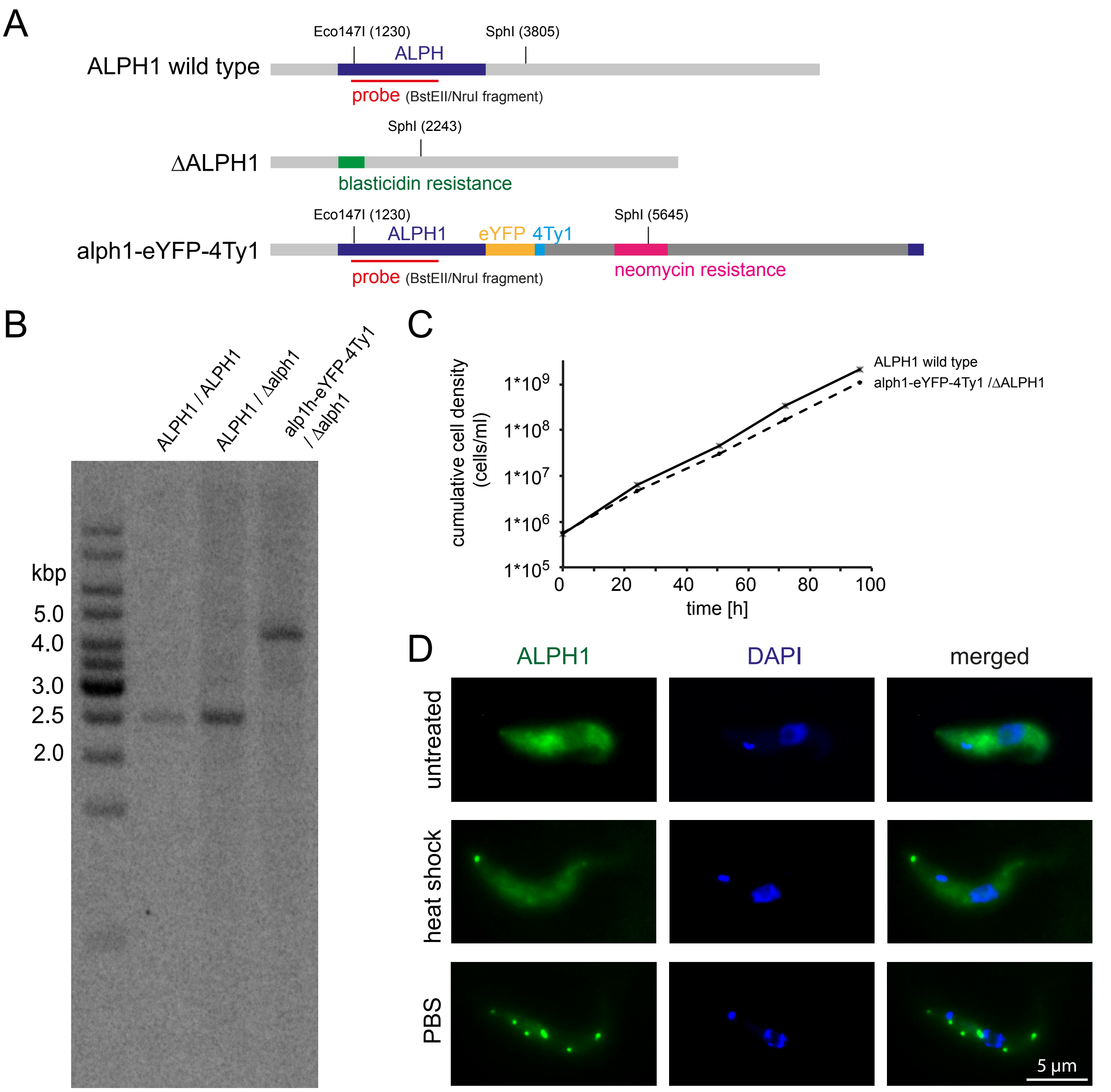

Figure S6: A cell line entirely dependent on Tbalph1-eYFP4Ty1 is viable
Data are shown for one out of 4 clonal cell lines.

A) To scale schematics of the genomic locus of TbALPH1 (top), the genomic locus of TbALPH1 after ALPH1 was replaced by a blasticidin resistance cassette (middle) and the TbALPH1 genomic locus after endogenous, C-terminal tagging of ALPH1 with eYFP-4Ty1 ALPH1 (bottom). Restriction enzyme recognition sites and positions of probes that are relevant for the Southern blot in B are indicated.

B) Southern blot: Genomic DNA of wild type cells (ALPH1/ALPH1), cells with one ALPH1 allele deleted (ALPH1/ Δalph1) and cells with one ALPH1 allele deleted and the second allele modified by a plasmid that adds a C-terminal eYFP-4Ty1 tandem tag (alp1h-eYFP-4Ty1 / Δalph1) was digested with Eco147I and SphI and the resulting fragments were detected by a Southern blot probed for part of the ALPH1 open reading frame (see A). The Southern blot shows the correct genotype of the alp1h-eYFP-4Ty1 / Δalph1 cell line: the cell line has no wild type ALPH1.

C) Growth curve: growth of wild type cells and alp1h-eYFP-4Ty1 / Δalph1 cells was measured. There is no major reduction in growth in the alp1h-eYFP-4Ty1 / Δalph1 cell line in comparison to wild type cells.

D) alph1-eYFP-4Ty1 / Δalph1 cells were examined by fluorescence microscopy, either untreated, after heat shock (2 h at 41°C) or after PBS starvation (2 hours). In untreated cells, only a minor proportion of alph1-eYFP-4Ty1 is localised at the posterior pole. Both heat shock and PBS starvation causes accumulation of alph1-eYFP-4Ty1 to the posterior pole, PBS starvation also cause localisation to starvation stress granules. Thus, the localisation of alph1-eYFP-4Ty1 in the absence of wild type ALPH1 is identical to its localisation with the wild type ALPH1 present.
