# Supplementary material for: The ApaH-like phosphatase TbALPH1 is the major mRNA decapping enzyme of trypanosomes
Source: PLoS Pathog. 2017 Jun 19;13(6):e1006456. doi: 10.1371/journal.ppat.1006456 (PMC5491325; doi:10.1371/journal.ppat.1006456)
Supplement: S7 Fig — (PDF) [file ppat.1006456.s007.pdf]

Figure S7

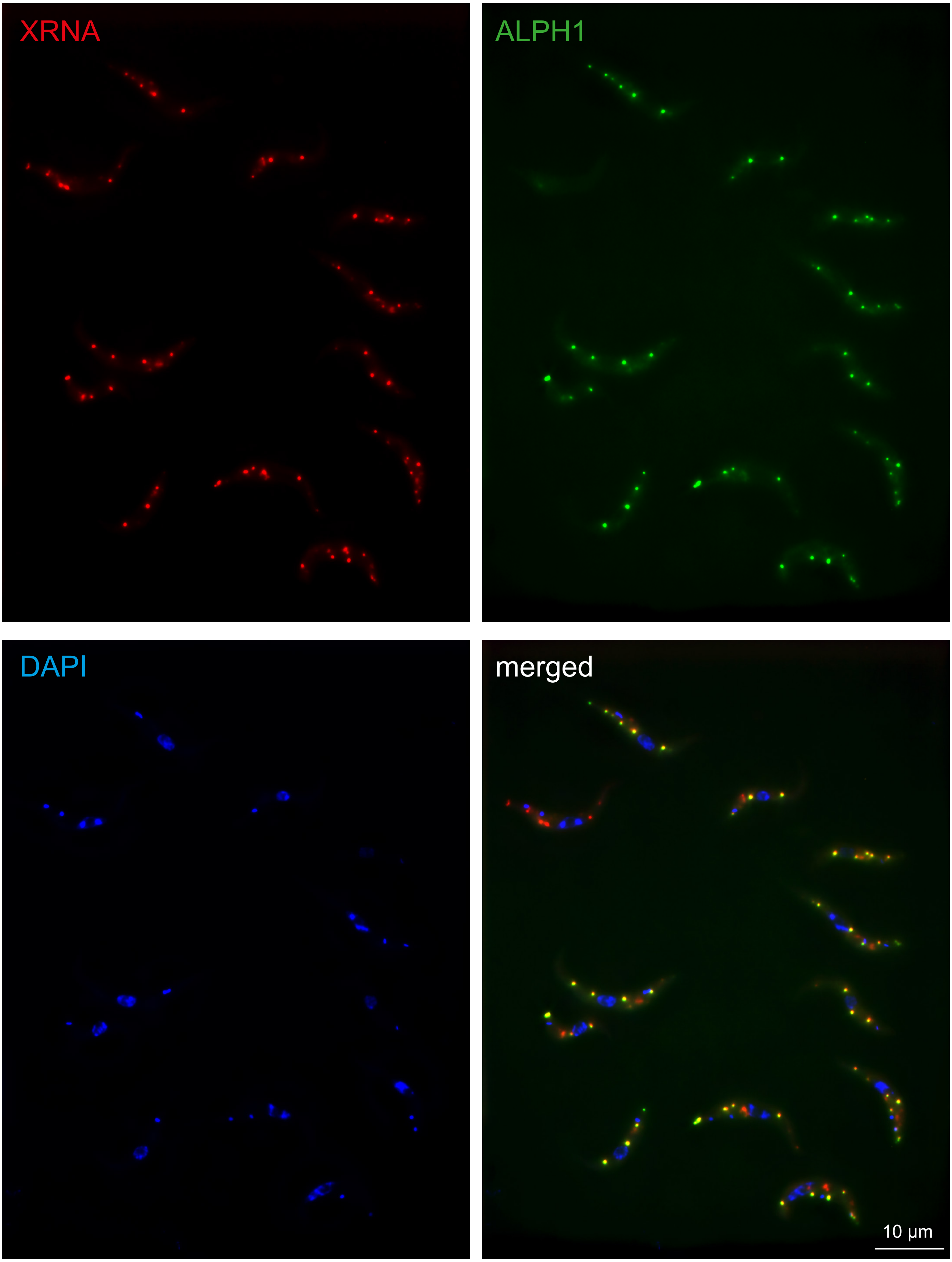

Figure S7: Cells co-expressing XRNA-mChFP and ALPH1-eYFP from endogenous loci were treated with starvation (PBS). Note that the expression levels of XRNA-mChFP are very low and thus, the autofluorescence of the lysosome becomes partially visible.
