# Supplementary material for: The ApaH-like phosphatase TbALPH1 is the major mRNA decapping enzyme of trypanosomes
Source: PLoS Pathog. 2017 Jun 19;13(6):e1006456. doi: 10.1371/journal.ppat.1006456 (PMC5491325; doi:10.1371/journal.ppat.1006456)
Supplement: S8 Fig — (PDF) [file ppat.1006456.s008.pdf]

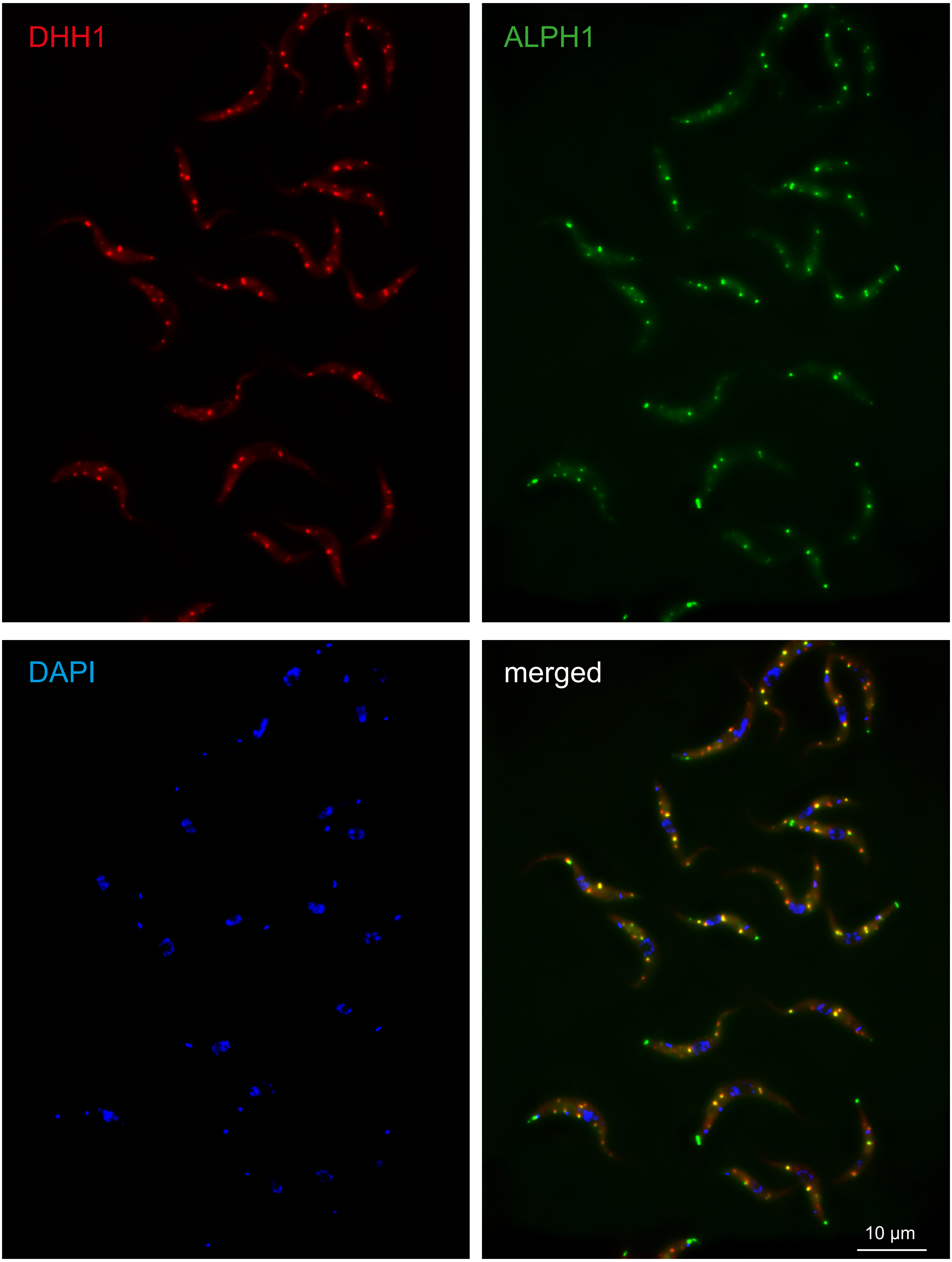

Figure S8: Cells co-expressing mChFP-DHH1 and ALPH1-eYFP from endogenous loci were treated with starvation (PBS).
