# Supplementary material for: The ApaH-like phosphatase TbALPH1 is the major mRNA decapping enzyme of trypanosomes
Source: PLoS Pathog. 2017 Jun 19;13(6):e1006456. doi: 10.1371/journal.ppat.1006456 (PMC5491325; doi:10.1371/journal.ppat.1006456)
Supplement: S9 Fig — (PDF) [file ppat.1006456.s009.pdf]

Figure S9

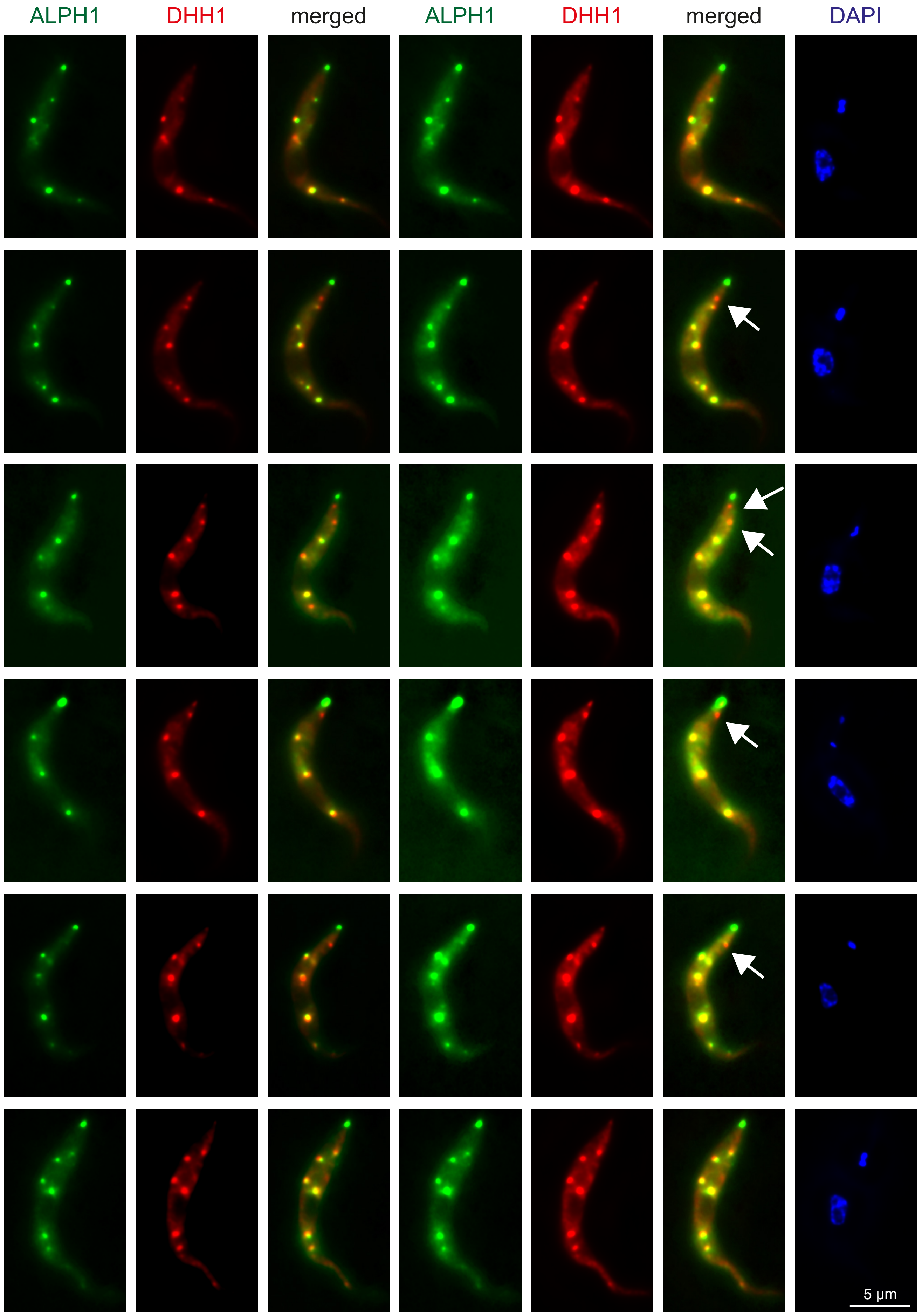

Figure S9: Examples images of cells co-expressing ALPH1-eYFP and mChFP-DHH1 from the endogenous locus. For the red and green fluorescence, images are shown at two different levels of brightness. Starvation stress granules that lack ALPH1 are marked with an arrow; these are mostly at the posterior end of the cell.
