# Supplementary material for: The ApaH-like phosphatase TbALPH1 is the major mRNA decapping enzyme of trypanosomes
Source: PLoS Pathog. 2017 Jun 19;13(6):e1006456. doi: 10.1371/journal.ppat.1006456 (PMC5491325; doi:10.1371/journal.ppat.1006456)
Supplement: S10 Fig — (PDF) [file ppat.1006456.s010.pdf]

Figure S10

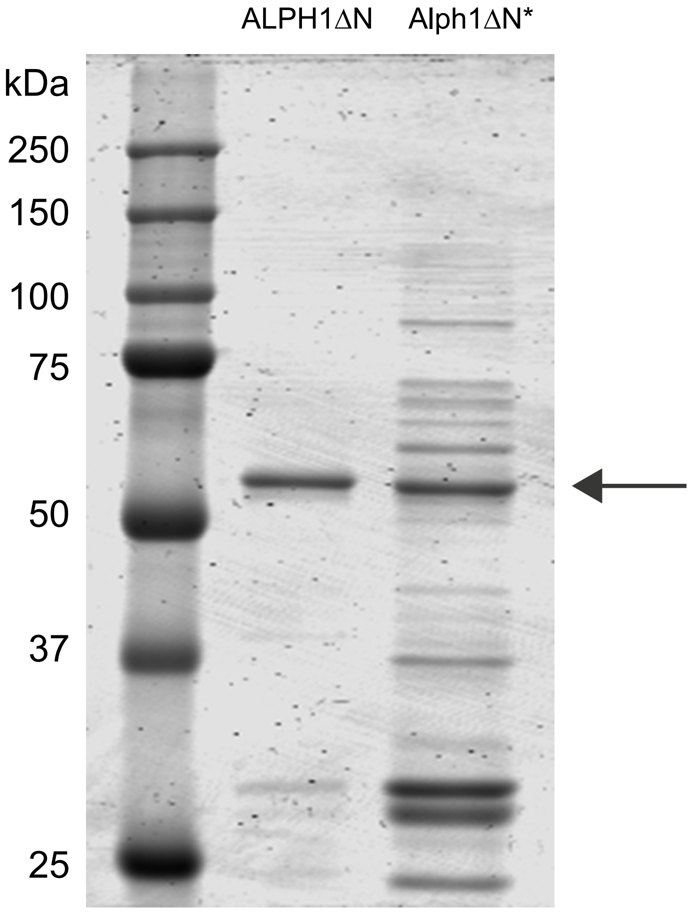

Figure S10: Coomassie stained gel loaded with 1 µl and 10 µl of ALPH1ΔN and Alph1ΔN* (arrow), purified from *E. coli*.The expected molecular weight of the histidine fusion protein is 59.6 kDa. Note that Alph1ΔN* could not be expressed to the same high levels as the wild type protein and contains therefore a higher amount of contaminations. However, given that this protein served as a negative control, mainly to control that no bacterial contaminations are responsible for the decapping activity, this higher level of contaminations does not affect the interpretation of the data. For each decapping assay, 2 µl of wild type ALPH1ΔN and 20 µl of Alph1ΔN* were used. This corresponds to roughly 800 ng protein, when estimated from a BSA calibration curve (data not shown).
