# Supplementary material for: The ApaH-like phosphatase TbALPH1 is the major mRNA decapping enzyme of trypanosomes
Source: PLoS Pathog. 2017 Jun 19;13(6):e1006456. doi: 10.1371/journal.ppat.1006456 (PMC5491325; doi:10.1371/journal.ppat.1006456)
Supplement: S12 Fig — (PDF) [file ppat.1006456.s012.pdf]

Figure S12

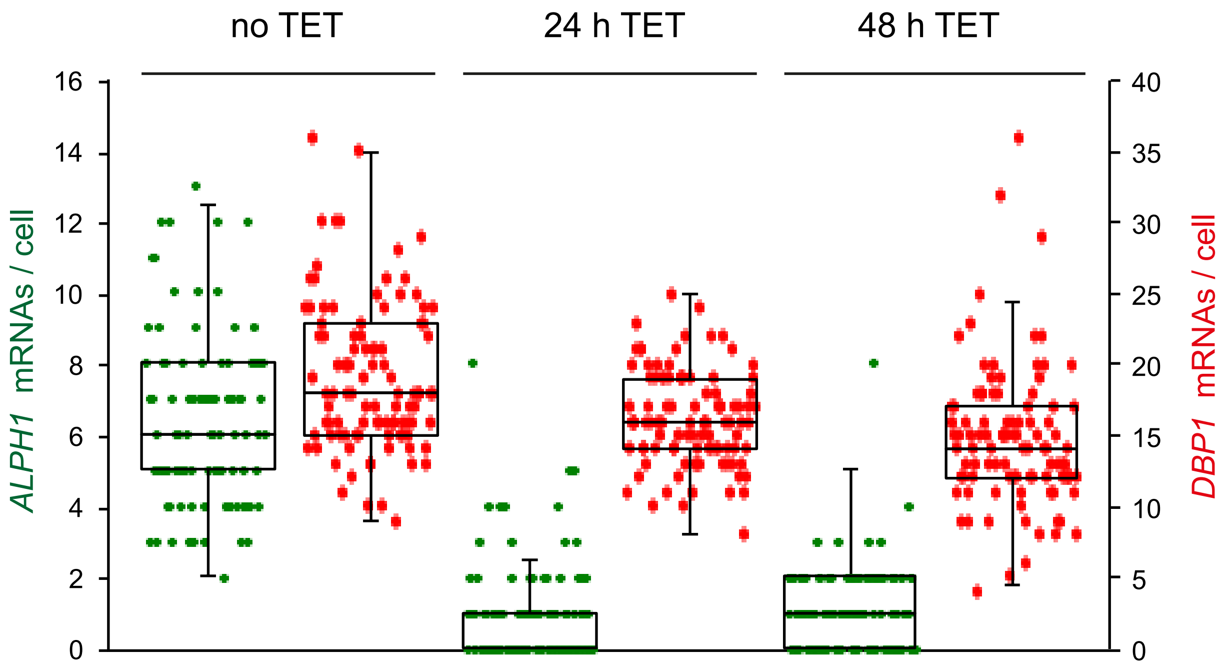

Figure S12: Reduction in the number of *ALPH1* mRNA molecules after RNAi depletion of ALPH1 (clone 2).

ALPH1 mRNA and *DBP1* mRNA (control) were visualized by dual colour single molecule mRNA FISH (Affymetrix®), using green (*ALPH1*) and red (*DBP1*) fluorescent Affymetrix probe sets. The number of mRNA molecules per cell was quantified after 0, 24 and 48 hours of ALPH1 RNAi induction. Data are presented as box plot (waist is median; box is IQR; whiskers are ±1.5 IQR; only the smallest and largest outliers are shown; n = 100 for each time-point); the number of mRNAs from the individual cells is also presented as green (*ALPH1*) or red (*DBP1*) dots.
